# Exceptional hydrogen storage achieved by screening nearly half a million metal-organic frameworks

**DOI:** 10.1038/s41467-019-09365-w

**Published:** 2019-04-05

**Authors:** Alauddin Ahmed, Saona Seth, Justin Purewal, Antek G. Wong-Foy, Mike Veenstra, Adam J. Matzger, Donald J. Siegel

**Affiliations:** 1 Mechanical Engineering Department, University of Michigan, Ann Arbor, MI 48109 United States; 2 Department of Chemistry, University of Michigan, Ann Arbor, MI 48109 United States; 3 Ford Motor Company, Research and Advanced Engineering, 1201 Village Rd., Dearborn, MI 48121 United States; 4 Materials Science & Engineering, University of Michigan, Ann Arbor, MI 48109 United States; 5 Applied Physics Program, University of Michigan, Ann Arbor, MI 48109 United States; 6 University of Michigan Energy Institute, University of Michigan, Ann Arbor, MI 48109 United States

## Abstract

Few hydrogen adsorbents balance high usable volumetric and gravimetric capacities. Although metal-organic frameworks (MOFs) have recently demonstrated progress in closing this gap, the large number of MOFs has hindered the identification of optimal materials. Here, a systematic assessment of published databases of real and hypothetical MOFs is presented. Nearly 500,000 compounds were screened computationally, and the most promising were assessed experimentally. Three MOFs with capacities surpassing that of IRMOF-20, the record-holder for balanced hydrogen capacity, are demonstrated: SNU-70, UMCM-9, and PCN-610/NU-100. Analysis of trends reveals the existence of a volumetric ceiling at ∼40 g H_2_ L^−1^. Surpassing this ceiling is proposed as a new capacity target for hydrogen adsorbents. Counter to earlier studies of total hydrogen uptake in MOFs, usable capacities in the highest-capacity materials are negatively correlated with density and volumetric surface area. Instead, capacity is maximized by increasing gravimetric surface area and porosity. This suggests that property/performance trends for total capacities may not translate to usable capacities.

## Abstract (web summary)

Considering the large number of existing synthesised and hypothesised metal-organic frameworks, determining which materials perform best for given applications remains a challenge. Here, the authors screen the usable hydrogen uptake capacities of nearly 500,000 MOFs, and find that three frameworks outperform the current record-holder.

## Introduction

Hydrogen is a promising vehicular fuel due to its high specific energy, renewability, and its ability to be produced and oxidized without CO_2_ emissions^1–3^. However, due to the low volumetric density of H_2_ gas, efficient and cost-effective storage of hydrogen remains a challenge^1–3^. To overcome this challenge, storage in solid adsorbents has received significant attention as an alternative to compression in high-pressure tanks^1–4^. Adsorbents have the potential to match or surpass the capacities typical of physical storage systems, while doing so at lower pressures and with the potential to reduce cost^1^.

Metal-organic frameworks (MOFs) are perhaps the most intensively-researched hydrogen adsorbents^1,5–17^. Microporous crystalline MOFs are formed by the self-assembly of inorganic metal clusters and organic linkers^18^. The many possible variations of these building blocks allow MOFs to exhibit a wide range of properties, some of which (e.g., surface area) are unmatched by other materials^5,19–23^. Although this design flexibility allows for the tuning of MOF properties, it also complicates the identification of optimal compositions, because the parameter space that must be searched is very large^1^.

Computational methods^24,25^ have been of great value in accelerating this search^7,11,16,17,26–36^. In the case of hydrogen storage, high-throughput calculations have assisted in the identification of MOFs with the potential to achieve high capacities. These techniques also allow for the identification of property-performance trends, resulting in design guidelines^37–39^. Table 1 summarizes recent high-throughput studies of hydrogen storage in MOFs. These studies have examined real MOFs (i.e., based on crystal structures of synthesized compounds), and larger collections of hypothetical compounds, which are generated computationally^6,17,33–35^. For example, a recent study by the present authors identified MOFs that simultaneously exhibit high volumetric and gravimetric hydrogen densities from a database of 5309 real compounds^1^. Promising MOFs were identified using Grand Canonical Monte Carlo (GCMC) calculations employing the pseudo-Feynman–Hibbs interatomic potential^1,40–42^. Consistent with the computational predictions, IRMOF-20 was demonstrated experimentally to exhibit an uncommon combination of high usable volumetric (UV) and gravimetric capacities^1^. Importantly, the measured capacities exceeded those of the benchmark compound MOF-5^40,43^, the previous record-holder for combined volumetric/gravimetric performance^1^.

**Table 1:** Summary of recent high-throughput calculations of hydrogen storage in MOFs

Database	Number of MOFs screened	H_2_ storage condition	Literature
CoRE+UM (real)	5,309	Usable: (77 K, 100 bar) → (77 K, 5 bar)	Ahmed et al. (2017)^1,8,24^
		Usable: (77 & 298 K, 100 bar) → (77 & 298 K, 1 bar)	Thornton et al. (2017)^11,24^
Northwestern (hypothetical)	137,953	Usable: (77 & 298 K, 100 bar) → (77 & 298 K, 1 bar)	Thornton et al. (2017)^11,32^
		Usable: (77 K, 100 bar) → (77 K, 2 bar)	Bobbitt et al. (2016)^16,32^
		Total: 1, 50, and 100 atm at 77 K	Gomez et al. (2014)^67,32^
ToBaCCo (hypothetical)	13, 512	Total: 100 bar at 130, 200, and 243 K	Colón et al. (2017)^6,17^
		Usable: (77 K, 100 bar) → (77 K, 5 bar)	Gómez-Gualdrón et al. (2016)^6,17^
Mg-MOFs (hypothetical)	18,383	Usable: (243 K, 100 bar) → (243 K, 2 bar)	Colón et al. (2014)^55^
		Total: 243 K & 100 bar	
UM (real)	~4,000	Total: 77 K & 35 bar	Goldsmith et al. (2013)^8^

The demonstration of IRMOF-20 raises the possibility that other high-capacity MOFs may exist. Indeed, many other databases of MOFs exist beyond those examined in ref. ^1^. Nevertheless, to our knowledge a systematic assessment of hydrogen capacities across all published databases of real and hypothetical MOFs has not been reported. The present study expands upon prior work by casting a significantly wider net: by assembling a database of databases nearly 500,000 real and hypothetical MOFs (Table 2) have been examined computationally. The most promising materials identified computationally were subsequently synthesized and characterized experimentally. Importantly, three MOFs with usable capacities surpassing that of IRMOF-20 have been demonstrated: SNU-70, UMCM-9, and PCN-610/NU-100. These materials establish a new high-water mark for usable hydrogen capacities in MOFs under physisorptive, pressure-swing (PS) conditions. Analysis of trends across the database reveals the existence of a volumetric ceiling at ~40 g-H_2_ L^−1^. This ceiling highlights the need to develop new adsorbents that are specifically constructed to exhibit larger volumetric capacities. Counter to earlier studies of total hydrogen uptake in MOFs^8^, usable capacities in the highest performing materials identified here were found to be negatively correlated with density and volumetric surface area (VSA). Instead, usable capacities are maximized by increasing gravimetric surface area (GSA) and porosity. These observations suggest that property-performance trends identified for total capacities may not apply to usable capacities.

## Results

### Computational screening

**Fig. 1:**
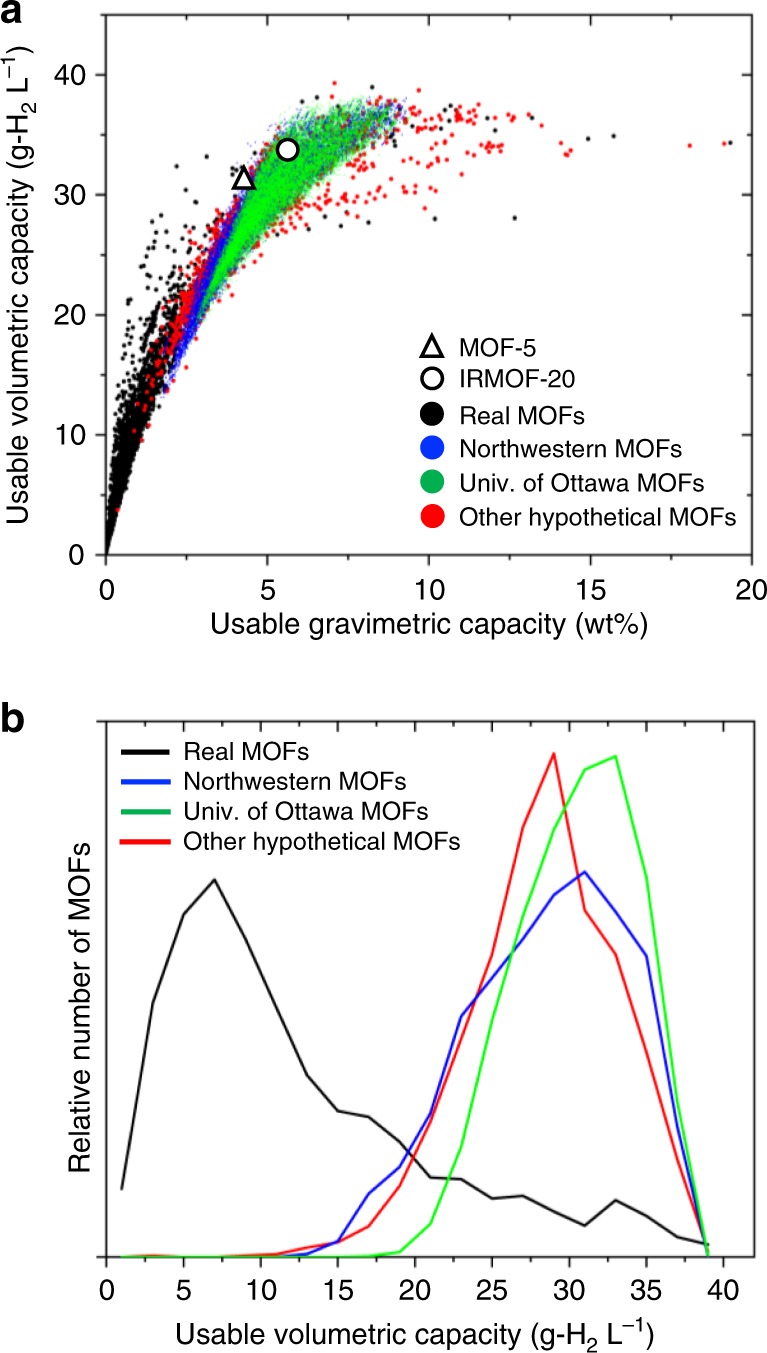
High-throughput screening of MOFs. **a** Calculated usable hydrogen capacities of 43,777 MOFs compared to the measured capacity of IRMOF-20 (5.7 wt.%, 33.4 g-H_2_ L^−1^) and MOF-5 (4.5 wt.% and 31.1 g-H_2_ L^−1^). **b** Relative number of MOFs as a function of usable volumetric capacity and their originating database

Fig. 1a shows the calculated UV capacities of 43,777 MOFs examined with GCMC as a function of their usable gravimetric (UG) capacities. These MOFs were down-selected using an initial screen based on crystallographic properties and the Chahine rule^8^ (see Methods) from a database of 493,458 MOFs described in Table 2 and in Supplementary Tables 1–10. Capacities were evaluated assuming a pressure swing between 100 bar and 5 bar at 77 K. These capacities are compared to that of IRMOF-20, which was identified in prior work as having the best combination of gravimetric & volumetric capacities under these pressure-temperature conditions^1^, and to MOF-5, a benchmark MOF adopted by the Hydrogen Storage Engineering Center of Excellence (HSECoE)^3,43^. Irrespective of the database used, Fig. 1a shows that volumetric capacities increase monotonically with increasing gravimetric capacity up to ~10 wt.%, at which point volumetric performance plateaus below ~40 g-H_2_ L^−1^. A similar trend was reported in our earlier study, which examined a smaller database containing 5309 real MOFs^1^. Considering first the performance of the real MOFs^8,24,25^, only 102 compounds are predicted to exhibit usable capacities greater than that of IRMOF-20 on both a volumetric and gravimetric basis. Supplementary Table 3 lists the 50 highest-capacity MOFs ranked according to volumetric H_2_ density. Out of the identified compounds, ECOLEP (Cambridge Structural Database (CSD) refcode) exhibits the best UV performance, as well as an appealing UG capacity: 39 g-H_2_ L^−1^ & 8.2 wt.%. On the other hand, the highest UG capacity (irrespective of UV) is predicted for MOF-399 (CSD refcode BAZGAM) : 34.3 g-H_2_ L^−1^ & 19.3 wt. %.

Regarding the relative performance of the different databases, the capacities of the real MOFs mostly fall within a lower capacity region between 0–5 wt. % and 0–25 g-H_2_ L^−1^. This behavior is again consistent with our prior study^1^. In contrast, compounds drawn from the hypothetical MOF databases can exhibit much higher capacities. For example, the Univ. of Ottawa database contains the largest number of MOFs that exceed IRMOF-20 in terms of UV and UG capacities; 3581 MOFs surpass this threshold. Similarly, 2154 and 222 MOFs from the Northwestern and remaining (combined) hypothetical MOF databases, respectively, show promise for achieving capacities in excess of IRMOF-20^32,44^. Supplementary Tables 4–10 list the 20 highest capacity MOFs from each database in Table 2 that exceed the volumetric capacity of IRMOF-20. The synthesizability of these hypothetical MOFs remains unclear.

**Table 2:** Details of the MOF database

Database	No. of MOFs	No. with zero surface area	Capacity exceeds MOF-5	Exceeds IRMOF-20
Real MOFs:^8,24,25^ UM+CoRE+CSD	15,235	2950	405	102
Mail-order^35^	112	4	30	19
In silico deliverable^34^	2816	154	27	6
In silico surface^56^	8, 885	283	236	77
MOF-74 analogs^58^	61	0	0	0
ToBaCCo^17^	13,512	214	135	72
Zr-MOFs^27^	204	0	126	35
Northwestern^32^	137,000	30,160	4397	2154
Univ. of Ottawa^44^	315,615	32,993	7612	3581
In-house	18	0	18	13
Total	493,458	66,758	12,986	6059

Details of the MOF database, including the number of MOFs in a given database, the number with negligible internal surface area, and the number of compounds identified by GCMC that exceed the usable, pressure-swing capacities of MOF-5 and IRMOF-20. Additional details can be found in Methods Section, Supplementary Methods, Supplementary Figure 1 and Supplementary Tables 1-10

Figure 1b shows the relative number of MOFs having a given UV capacity as a function of the MOF database in which they are found. For the real MOFs the probability distribution is asymmetric and peaked at low capacities: the number of MOFs increases rapidly with UV up to a maximum around 7 g-H_2_ L^−1^, but then exhibits a long tail extending out to nearly 40 g-H_2_ L^−1^. In contrast, the distributions for the hypothetical MOF databases show opposite behavior: these distributions are skewed to higher UV, with maxima between 28 to 32 g-H_2_ L^−1^. As expected from Fig. 1a, the distributions in Fig. 1b all approach zero for UV capacities approaching 40 g-H_2_ L^−1^. It is unclear if this volumetric ceiling represents an intrinsic limitation of MOFs, or simply reflects the design decisions made in the assembly of the MOFs present in these databases. A maximum materials-level UV capacity of 40 g-H_2_ L^−1^ could in principle surpass the DOE 2020 system-level target of 30 g-H_2_ L^−1^, assuming modest volumetric penalties associated with the system. Nevertheless, a MOF below this ceiling could achieve neither the 2025 (40 g-H_2_ L^−1^) nor the Ultimate targets (50 g-H_2_ L^−1^)^4,45^. Thus it is suggested that MOF designs that specifically target higher UV be pursued.

### MOF Synthesis

**Fig. 2:**
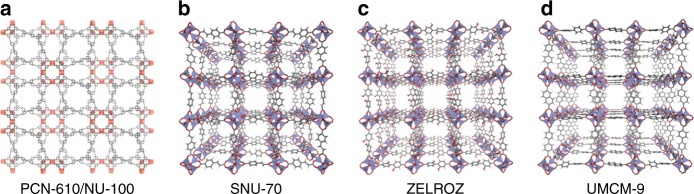
Crystal structures of MOFs whose hydrogen uptake was assessed experimentally following their identification by computational screening. **a** PCN-610/NU-100, **b** SNU-70, **c** ZELROZ, and **d** UMCM-9 (C: gray, H: white, O: red, Cu: orange and Zn: blue)

Several of the highest-capacity MOFs predicted by GCMC calculations were targeted for synthesis. These MOFs were selected based on their perceived stability and synthetic accessibility, in addition to their potential to exhibit high capacities exceeding that of IRMOF-20 and MOF-5. These considerations initially resulted in the selection of PCN-610/NU-100 (refcodes  HABQUY/GAGZEV), SNU-70/ MOF-5_cooh_2_567_1_basic_opt, and the MOF with CSD refcode ZELROZ. (Although ECOLEP was also predicted to have the highest UV capacity overall, it could not be synthesized in a phase-pure form.) Fig. 2 illustrates the structures of these MOFs. PCN-610/NU-100 is a well-known MOF which was identified from the database of real MOFs^8,24,25^. To our knowledge its usable capacity for the pressure range 5–100 bar has not been reported. MOF-5_cooh_2_567_1_basic_opt is a hypothetical MOF identified from the mail-order MOF database^35^. Analysis of its structure revealed that is an ordered variant of the known MOF, SNU-70 (GEBPEK)^46^. ZELROZ contains 2,2′-dihydroxy-[1,1′-biphenyl]-4,4′-dicarboxylate linkers and is isoreticular with MOF-5 and IRMOF-20^47^. Although it has a higher projected usable capacity than IRMOF-20, it could not be realized in a fully activated form; only ~70% of the calculated GSA was achieved.

Further, the calculations also identified non-interpenetrated IRMOF-10 as having higher usable capacities than IRMOF-20 (Supplementary Table 1). This MOF is an unfunctionalized variant of ZELROZ, and also contains the 1,1′-biphenyl-4,4′-dicarboxylate linker^48^. Unfortunately, the structure of IRMOF-10 is missing from the CSD, likely due to the presence of disorder. In addition, IRMOF-10 has never been obtained with high surface area, likely due to interpenetration or pore collapse during activation. As an alternative, a mixed linker-based approach was explored as a means to realize non-interpenetrated MOFs from 1,1′-biphenyl-4,4′-dicarboxylate linkers^49^. UMCM-9, containing 1,1′-biphenyl-4,4′-dicarboxylate and 2,6-naphthalenedicarboxylate linkers (Fig. 2), is known to have a non-interpenetrated framework with high experimental GSA. Its structure is, however, highly disordered and therefore is not present in the CSD. An ordered structure of the MOF was constructed for GCMC calculations, and favorable capacity predictions from these calculations prompted experimental investigation of its hydrogen storage capacity. The present calculations on UMCM-9 employed a structure developed from powder diffraction data^49^.

Table 3 summarizes the measured and calculated crystallographic properties of these MOFs. (N_2_ isotherms used in Brunauer-Emmett-Teller surface area measurements are shown in Supplementary Figures 2–4.) Correlations between these crystallographic properties and usable capacities are discussed below.

**Table 3:** Measured and calculated crystallographic properties of high-capacity MOFs examined in this study

MOF	Gravimetric surface area (m^2^ g^−1^) Expt./Calc.	Volumetric surface area (m^2^ cm^−3^) Calc.	Pore volume (cm^3^ g^−1^) Calc.	Void fraction Calc.
MOF-5	3512/3563	2172	1.36	0.81
IRMOF-20	4073/4127	2000	1.65	0.84
SNU-70	4944/4756	1905	2.14	0.86
UMCM-9	5039/4847	1805	2.31	0.86
PCN-610/NU-100	6050/5777	1603	3.17	0.88

### Hydrogen capacity—pressure swing

**Fig. 3:**
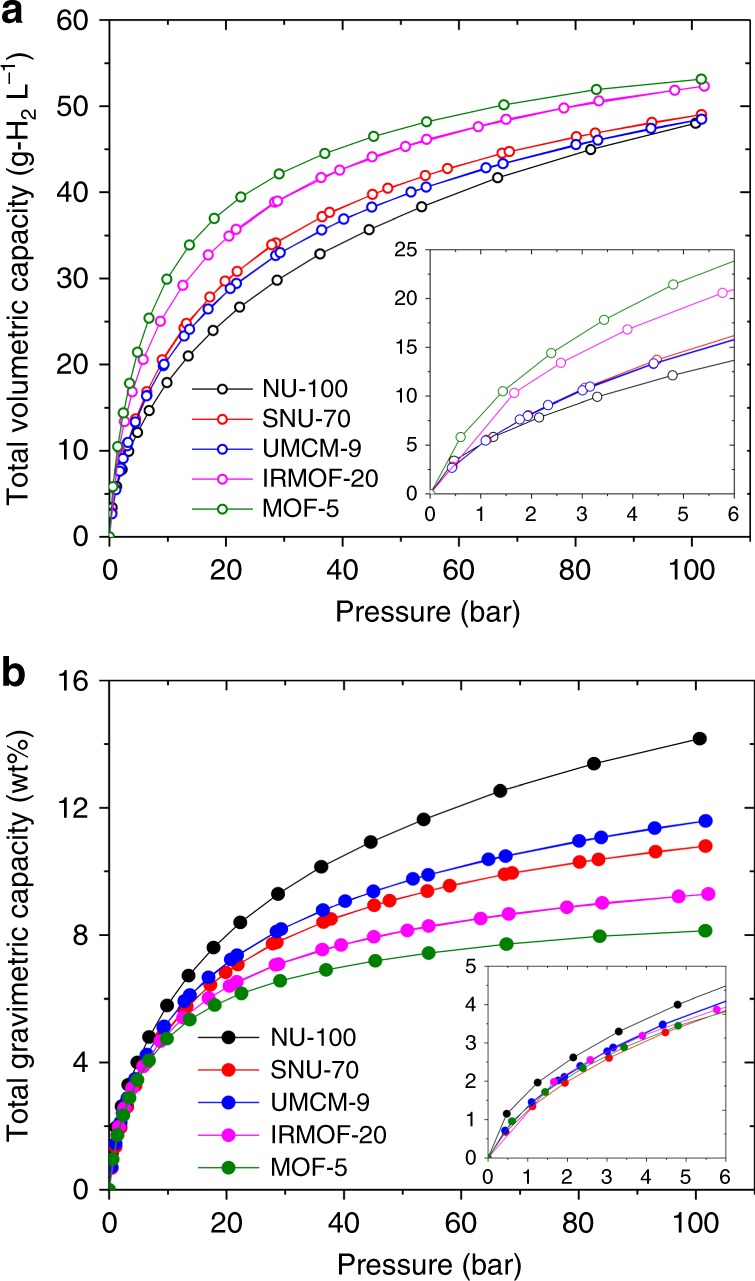
H_2_ adsorption isotherms. Measured total (**a**) volumetric and (**b**) gravimetric H_2_ adsorption isotherms of NU-100, SNU-70, and UMCM-9 at 77 K. For comparison, isotherms (ref. ^1^) for the two benchmark MOFs, MOF-5 and IRMOF-20, are also shown. Inset plots illustrate capacities at low pressure

Figure 3 shows the measured H_2_ adsorption isotherms of PCN-610/NU-100, SNU-70, and UMCM-9 at 77 K. Isotherms for IRMOF-20 and MOF-5 are also shown for comparison^1^. A comparison between the measured and calculated isotherms are shown in Supplementary Figures 5–7. In general, good agreement is achieved between the measurements and calculations.

**Fig. 4:**
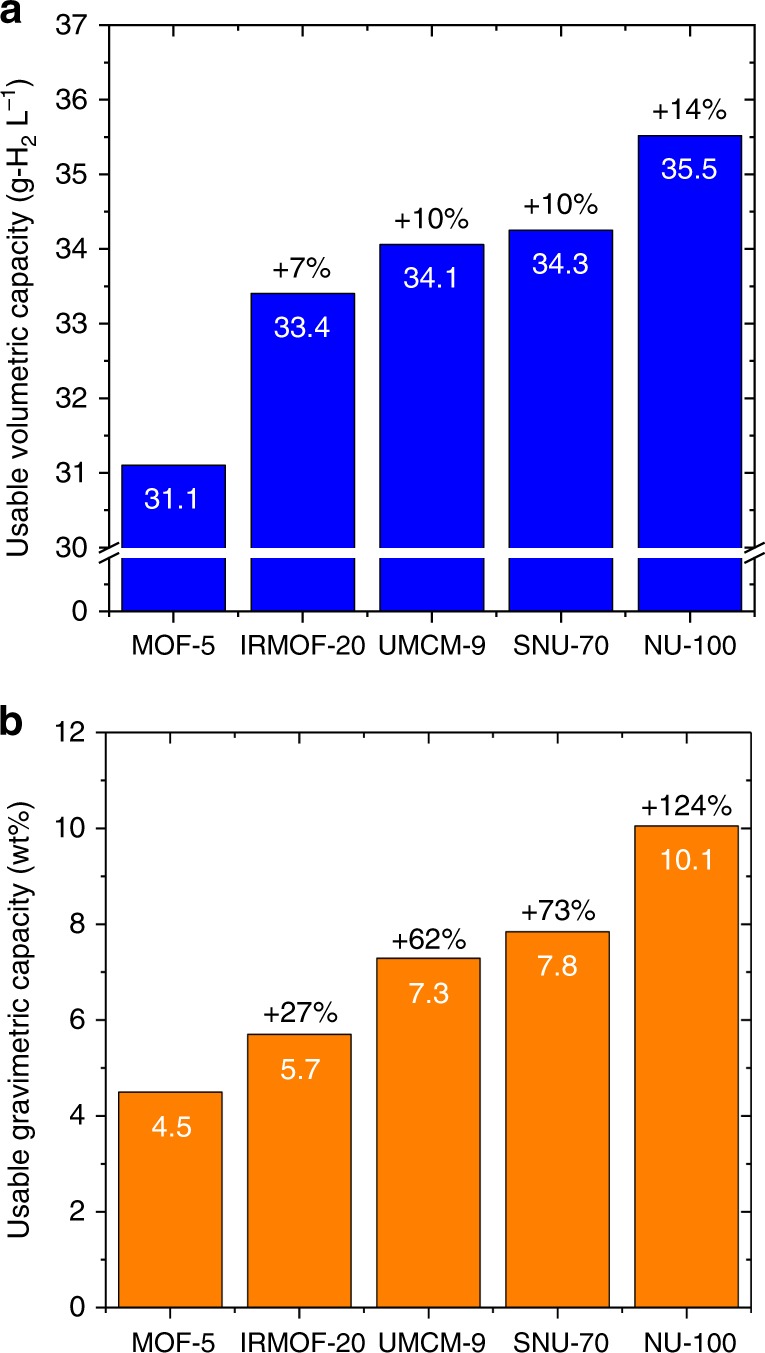
Measured usable H_2_ storage capacities of MOFs. **a** Volumetric basis and **b** gravimetric basis. Capacities are reported for an isothermal pressure swing at 77 K between 5 and 100 bar. Data for MOF-5 and IRMOF-20 are taken from ref. ^1^. Percentages listed at the top of each bar correspond to improvements over MOF-5

Regarding the total volumetric (TV) capacities of these MOFs, Fig. 3a shows that MOF-5 and IRMOF-20 are, respectively, the two highest capacity MOFs for all pressures greater than ~1 bar. SNU-70, UMCM-9, and PCN-610/NU-100 all exhibit lower total capacities. Nevertheless, these latter three MOFs are superior on a usable basis, as shown in Fig. 4a. UV capacities range from 34.1 to 35.5 g-H_2_ L^−1^, which exceed those of MOF-5 and IRMOF-20 (31.1 and 33.4 g-H_2_ L^−1^, respectively). To our knowledge these usable capacities are the highest demonstrated for an adsorbent under these conditions. The superior usable performance of these MOFs can be understood by recalling that usable capacity is defined as the difference in total uptake at 100 bar (filled tank condition) and at 5 bar (empty tank). A significantly lower total uptake at 5 bar (compared to MOF-5 & IRMOF-20), combined with a modest difference in uptake at 100 bar, results in a larger usable capacity.

Regarding total gravimetric (TG) capacities, Fig. 3b shows that the trio of MOFs examined here outperform the benchmark MOF-5 and IRMOF-20 compounds for pressures exceeding 10 bar. Below this pressure (Fig. 3b inset) all five MOFs behave similarly, while at high pressures PCN-610/NU-100 is clearly superior. The similar low-pressure total capacities imply that that usable capacities will track with the capacities observed at high pressure. Fig. 4b confirms that this is the case: usable gravimetric capacities range from 7.3 (SNU-70) to 10.1 (PCN-610/NU-100), surpassing those of MOF-5 (4.5 wt.%) and IRMOF-20 (5.7 wt.%).

These data show that PCN-610/NU-100 has the highest usable PS capacity overall, on both a gravimetric and volumetric basis. We previously reported that MOFs with high TG capacities typically exhibit TV capacities that are unexceptional^1^. Consistent with that trend, Fig. 3a shows that the TG capacity of PCN-610/NU-100 at 100 bar is the largest of the five MOFs examined, yet its TV capacity is the lowest. Nevertheless, on a usable basis PCN-610/NU-100 emerges as a promising MOF due to its low uptake at 5 bar. Thus, we conclude that total capacities can be a poor indicator of useable capacity under PS conditions.

How do these materials-level capacities compare to the DOE system-level targets? Due of penalties associated with the mass and volume of the storage system, to meet these targets a storage material will need to exceed the desired system capacity considerably. The usable gravimetric capacity of PCN-610/NU-100 exceeds the 2020 target^4,45^ (4.5 wt.%) by 124% and exceeds the Ultimate target^4,45^(6.5 wt.%) by 55%. These values provide hope that a PCN-610/NU-100-based storage system could meet the targets, even when accounting for the mass of the system. In contrast, meeting the volumetric targets will be more challenging. PCN-610/NU-100 exceeds the DOE’s 2020 system-level target^4,45^ by only 18%, and falls 29% below the Ultimate targets^4,45^. Factoring in the volume of components other than the storage medium, we conclude that a PCN-610/NU-100-based system is either unlikely (in the case of the 2020 target^4,45^) or unable (Ultimate target^4,45^) to satisfy the DOE goals. This shortcoming points to the importance of emphasizing volumetric hydrogen density in the design of new storage materials^50^.

### Correlations and trends

**Fig. 5:**
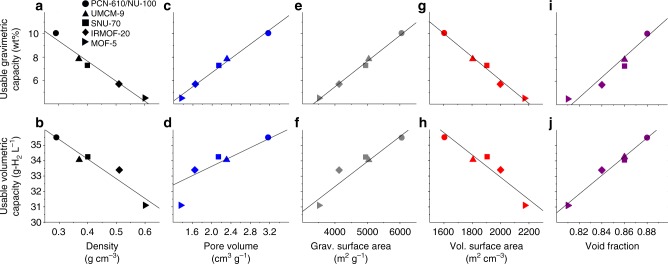
Relationship between five crystallographic properties and the usable capacities of the highest-capacity MOFs examined in the present study. Capacities are evaluated assuming an isothermal pressure swing between 5 and 100 bar at 77 K. (**a**, **c**, **e**, **g**, **i**) usable gravimetric capacities, (**b**, **d**, **f**, **h**, **j**) usable volumetric capacities

Do the properties of the MOFs characterized here correlate with their capacities? Fig. 5 plots UV and UG capacities as a function of 5 crystallographic features: density (D), pore volume (PV), GSA & VSA, and void fraction (VF). Roughly linear relationships are observed in all cases. Moreover, the same MOFs that bound the capacity range examined in Fig. 4, MOF-5 and PCN-610/NU-100, also bound the range of each crystallographic property. Usable capacities are positively correlated with PV (Fig. 5c, d), GSA (Fig. 5e, f), and VF (5i–j). In contrast, capacities are inversely related to single crystal density (Fig. 5a, b) and volumetric surface area (Fig. 5g, h). Thus, under these conditions, high usable capacities are achieved by maximizing PV, GSA, and VF, while simultaneously minimizing density and volumetric surface area.

**Fig. 6:**
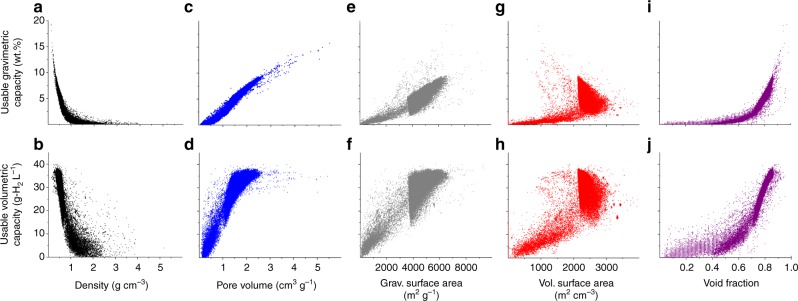
Usable capacities of 43,777 MOFs as a function of five crystallographic properties, assuming pressure-swing operation between 100 and 5 bar at 77 K. (**a**, **c**, **e**, **g**, **i**) gravimetric capacities, (**b**, **d**, **f**, **h**, **j**) volumetric capacities

Figure 6 examines these capacity-property trends more broadly across the set of ~43,000 MOFs examined with GCMC calculations. Turning first to the properties which were revealed in Fig. 5 to correlate positively with capacity (PV, GSA, and VF), Fig. 6 demonstrates that these same trends generally hold across the entire MOF dataset: maximizing PV, GSA, and VF maximizes UV and UG capacities. In contrast, Fig. 6 reveals that the inverse correlations that hold for D and VSA for the highest-capacity MOFs (Fig. 5) do not apply generally. For example, Fig. 5a, b suggests that capacity can be maximized by minimizing D. While this is indeed true for the narrow range of D examined in Fig. 5a, b (0.3 to 0.6 g cm^−3^), Fig. 6a, b shows that capacity decreases for densities outside of this range. Thus, a “sweet-spot” exists for D near 0.6 g cm^−3^. Similar behavior is observed for VSA: comparing Fig. 5g, h with Fig. 6g, h shows that VSA and usable capacity are in general related in a non-linear fashion. The linear relation observed in Fig. 5g, h is unique to the high-capacity MOFs examined here, and applies across a subset of VSA range from 1600 to 2200 m^2^ cm^−3^.

### Hydrogen capacity—temperature & pressure swing

Our analysis has thus far considered PS operation of the storage system. An important advantage of this scheme is its simplicity. Nevertheless, higher capacities can be achieved by adopting a more complex operating scheme. For example, the HSECoE has proposed an alternative operating scenario involving a combined temperature and pressure swing (TPS) from *T*_min_ = 77 K, *P*_max_ = 100 bar (filled) to *T*_max_ = 160 K, *P*_min_ = 5 bar (empty)^3,43,51,52^. By heating during desorption, the TPS approach increases capacity by minimizing the amount of H_2_ retained in the MOF in the low-pressure empty state^4^.

**Fig. 7:**
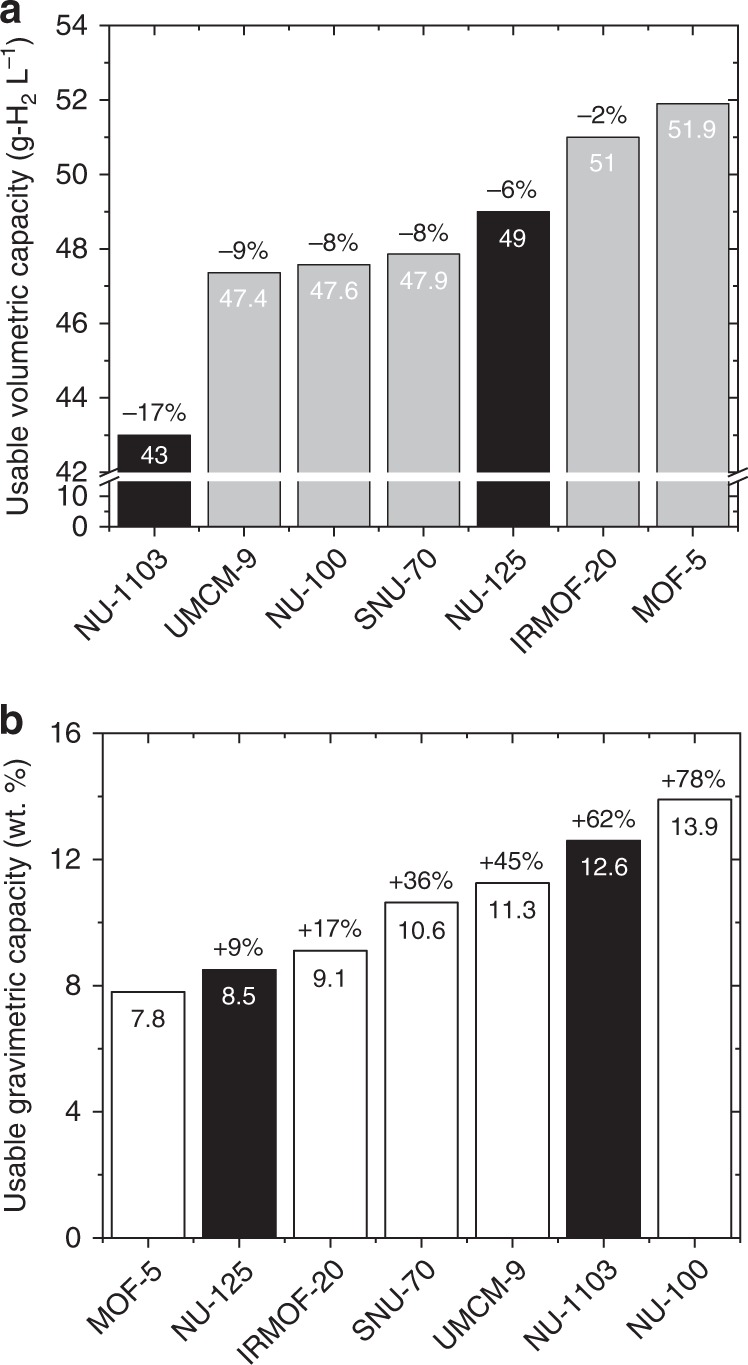
Comparison of measured usable H_2_ storage capacities of MOFs assuming temperature + pressure-swing operation between 100 bar-77 K and 5 bar-160 K. **a** Volumetric capacity, and **b** gravimetric capacity. Grey bars depict the performance of compounds reported in the present study or in the authors’ earlier report^1^. Black bars depict the performance of two high-capacity MOFs reported in ref. ^53^. Percentages on top of each bar depict performance relative to MOF-5

Figure 7 compares the measured usable TPS capacities of the present MOFs with the two highest-capacity MOFs identified in a recent study by García-Holley et al.^53^, NU-1103 and NU-125, and with the benchmark compounds MOF-5 and IRMOF-20^1^. Regarding volumetric capacities, Fig. 7a reveals that MOF-5 remains the top MOF (as previously noted)^1^, followed closely by IRMOF-20. The three MOFs identified in the present study as having high-capacities under PS conditions surpass the TPS capacity of NU-1103, but fall slightly below that of NU-125^53^. Assuming PS operation, the UV capacity of NU-125 is 24 g-H_2_ L^−1^, which is ~30% less than that of SNU-70 and UMCM-9, and ~32% less than that of PCN-610/NU-100. Additionally, the three MOFs synthesized here exhibit TPS volumetric capacities that surpass that of MFU-4*l* (47 g-H_2_ L^−1^), the top performing compound recently identified via machine learning screening of 50,000 MOFs^54^.

Regarding gravimetric TPS capacities, Fig. 7b shows that PCN-610/NU-100 is the top-performer, with a capacity of 13.9 wt.%. This exceptional gravimetric capacity contrasts with its volumetric performance, Fig. 7a, which is comparable to that of UMCM-9 and SNU-70. An even larger tradeoff in gravimetric/volumetric performance is evident for MOF-5, whose gravimetric capacity is the smallest of the 7 MOFs considered here.

More generally, Fig. 7 reveals that no single MOF studied here excels both gravimetrically and volumetrically under TPS operation. This situation differs from PS operation, where Fig. 4 shows that PCN-610/NU-100 is unambiguously the highest-capacity MOF. An ideal MOF would balance gravimetric and volumetric performance. In this regard, IRMOF-20 and SNU-70 both exhibit an appealing combination of volumetric and gravimetric TPS capacities. Furthermore, SNU-70, like UMCM-9, is derived from a commercially-available linker and may, therefore, provide cost advantages relative to MOFs requiring multistep synthesis of linkers.

In summary, a systematic assessment of hydrogen storage capacities in MOFs drawn from several published MOF databases has been presented. The goal was to identify MOFs that exhibit a balance of high volumetric and gravimetric hydrogen capacities under usable, physisorptive operating conditions. In total, nearly 500,000 compounds were screened computationally, and the most promising materials identified were synthesized and assessed experimentally. Three MOFs with usable, PS capacities surpassing that of IRMOF-20, the record-holder for balanced hydrogen capacity, were demonstrated: SNU-70 (identified from a hypothetical, ordered variant), UMCM-9, and PCN-610/NU-100. A similar analysis of the capacities of these MOFs under temperature+PS conditions also revealed them to be high-capacity materials.

Analysis of trends across the database points toward the existence of a volumetric ceiling at a capacity of ~40 g-H_2_ L^−1^. Surpassing this ceiling under usable, PS conditions is proposed as a new capacity target for hydrogen adsorbents. Counter to earlier studies of total hydrogen uptake in MOFs, usable capacities in the highest-capacity materials are negatively correlated with density and volumetric surface area. Instead, capacity is maximized by increasing GSA and porosity. These observations suggest that property-performance trends identified for total capacities may not translate to usable capacities.

## Methods

### Computational methods

A database of 493,458 MOF crystal structures, summarized in Table 2, was compiled by combining 11 published databases^8,17,24,25,27,34,35,44,55–57^. This meta-database consists of both real and hypothetical MOFs: 15,235 real MOFs were included from the UM^8^, CoRE^24^, and CSD^25^ databases; 478,205 hypothetical MOFs were aggregated from the Northwestern^32^, University of Ottawa^44^, mail-order^35^, in silico deliverable^34^, in silico surface^56^, MOF-74 analogs^58^, ToBaCCo^17^, and Zr-MOFs^27^ databases. Eighteen additional MOFs were included based on “in-house” designs. These latter compounds include hypothetical functionalized MOFs, as well as modeled crystal structures of real MOFs (such as UMCM-9) whose structures exhibit extensive disorder and are absent from the CSD. Additional details regarding the in-house database is available in Supplementary Table 1.

Crystallographic properties of all MOFs—single crystal density, gravimetric and volumetric surface areas, PV, VF, largest pore diameter, and pore limiting diameter—were calculated using the Zeo++ code (Supplementary Method 1.2)^59^.

As an initial screen, TG and TV H_2_ capacities were estimated at 77 K and 35 bar using the semi-empirical Chahine rule^8^. MOFs that surpassed the predicted capacity of MOF-5 under these conditions (TG > 8.4 wt.% and TV > 54.4 g-H_2_ L^−1^)^8^, amounting to 43,777 compounds, underwent further evaluation using GCMC calculations (see Supplementary Methods 1.3 & 1.4 for details), as described in an earlier study^60^. Usable capacities were calculated by GCMC assuming an isothermal PS between 5 and 100 bar at 77 K. MOFs predicted to have usable capacities exceeding MOF-5 (4.5 wt.% and 31.1 g-H_2_ L^−1^) and IRMOF-20 (5.7 wt.% and 33.4 g-H_2_ L^−1^) were identified and assessed for possible experimental characterization. Finally, the capacities for a subset of MOFs were predicted using GCMC under temperature+PS conditions between 77 K/100 bar and 160 K/5 bar. Additional details regarding these calculations can be found in Supplementary Mothods 1.3 & 1.4.

### Experimental methods

Promising MOFs identified by computation were synthesized and evaluated with respect to their hydrogen capacities. These included: SNU-70 (CSD refcode GEBPEK), PCN-610/NU-100 (CSD refcodes: HABQUY/GAGZEV), and UMCM-9 (Crystallographic Information File available in the Supplementary Information)^46,49,61,62^. With the exception of the PCN-610/NU-100 linker, all the metal salts and organic linkers were obtained from commercial sources. The linker for PCN-610/NU-100 was synthesized following the reaction scheme shown in Supplementary Figure 8^61,62^. Notably, this synthetic scheme involves only three steps (Supplementary Figures 8–11 show ligand characterization via NMR spectroscopy) leading to much higher yield of the final linker as compared to both reported procedures^61,62^. SNU-70 and PCN-610/NU-100 were activated by flowing supercritical (SC) CO_2_^63^. UMCM-9 was activated by successive exchanges of the guest solvent in the MOF pores with DMF, DCM, and *n*-hexane, and subsequently applying vacuum^64^.

UMCM-9 was synthesized following the literature procedure^49^. In five 60 mL glass jars with teflon-lined lids were added naphthalene-2,6-dicarboxylic acid (H_2_NDC, 0.0285 mg, 0.131 mmol), 1,1′-biphenyl-4,4′-dicarboxylic acid (H_2_BPDC, 0.0354 mg, 0.146 mmol), 6.7 mL of DEF, and 13.3 mL of N-methylpyrrolidone, and the solids were dissolved in the solvent mixtures by sonication. Subsequently, Zn(NO_3_)_2_·6H_2_O (0.235 g, 0.790 mmol) was added to the solution and the mixture was sonicated until transparent solutions were obtained. The reaction mixtures were heated to 85 °C for four days. Cubic crystals of UMCM-9 were formed at the inner surface of the vials along with minor amount of flocculent precipitate. After cooling to room temperature the mother liquor was decanted, the precipitate was removed by multiple DMF washes, and the crystals were collected together in a different vial. The MOF crystals were immersed in DMF for three days (washed several times with fresh DMF), then in dichloromethane for 18 h (washed with DCM, 20 mL × 8), and finally, in dry *n*-hexane for 12 h (washed with dry *n*-hexane 20 mL × 4). Subsequently, the solvent was decanted, the vial was placed in a vacuum chamber, and exposed to vacuum very slowly at room temperature. Finally, the material was activated under high vacuum (below 10^−4^ torr) for 26 h to yield clear pale yellow crystals (average yield 0.0523 g, 38%, based on H_2_NDC).

PCN-610/NU-100 was synthesized following the literature procedure^61^. 1,3,5-Tris[(1,3-carboxylic acid-5-(4-(ethynyl)phenyl)) ethynyl]benzene (LH_6_) (0.300 g, 0.32 mmol) and Cu(NO_3_)_2_·2.5H_2_O (0.600 g, 2.579 mmol) were dissolved in 36 mL DMF in a glass vial. Subsequently, 0.2 mL HBF_4_ was added to the solution, and the color of the solution turned teal. The solution was divided into thirty 4 mL vials (1.2 mL solution in each vial), and the vials were heated to 75 °C for 20 h. Teal colored octahedral crystals were formed at the bottom of the vial, which were collected together in a 60 mL jar, immersed in DMF for 1 day, and the supernatant liquid was replaced with fresh DMF (20 mL × 4) in this time. Subsequently, the MOF was immersed in ethanol for another day, and the liquid was replaced with fresh ethanol four times (20 mL × 4). The compound was then activated by flowing liquid CO_2_ at 2 mL min^−1^ flow rate for 1 h at room temperature, subsequently by supercritical CO_2_ at 2 mL min^−1^ flow rate for 2 h at 55 °C, and finally by supercritical CO_2_ at 1 mL min^−1^ flow rate for 6 h at 55 °C to result a purple solid (0.123 g, 34.4 % based on LH_6_).

SNU-70 was synthesized following the reported procedure with slight modification^46^. (E)-4-(2-Carboxyvinyl)benzoic acid (0.075 g, 0.390 mmol) and Zn(NO_3_)_2_·6H_2_O (0.150 g, 0.504 mmol) were dissolved in 25 mL DEF in a 60 mL glass jars with a teflon-lined lid. Six such reaction mixtures were heated to 105 °C for 12.5 h. At the end of this period, the glass jars were removed from the oven, and allowed to cool down to room temperature. Colorless cubic crystals (along with some fluffy precipitate) were formed at the bottom and the wall of the jars. The fluffy precipitate was removed from the MOF crystals by multiple wash with DMF. The remaining crystals were then collected together in a 60 mL glass vial, soaked in DMF and kept emerged for 2 d. The supernatant liquid was replaced with fresh DMF six times (20 mL each) in this time. The material was activated by SC CO_2_ flow by the same procedure as NU-100 (0.567 g, 51%).

MOFs were characterized by powder X-ray diffraction (PXRD) and surface areas were calculated from measured N_2_ isotherms following the recommendations by Rouquerol and co-workers^65^. Supplementary Figures 2–4 show the N_2_ isotherms used in Brunauer-Emmett-Teller (BET) surface area measurements. Comparisons of measured and simulated powder X-ray diffraction patterns (Supplementary Figures 12–14) confirm the crystallinity and phase purity of all three compounds.

Hydrogen adsorption and desorption measurements were performed using a manometric Sievert’s-type instrument (HPVA-200, Micromeritics Instrument Corporation)^66^. Additional details on MOF synthesis, activation, and characterization are provided in Supplementary Methods 4 & 5.

## Supplementary information

Supplementary Information

Peer Review File

## Data Availability

All calculated/measured crystallographic properties, adsorption isotherms, and characterization data sets generated and/or analyzed during the current study are available at the HyMARC Data Hub (https://datahub.hymarc.org) or from the corresponding author upon reasonable request. A portion of the MOF crystal structures used in generating the calculated data sets are accessible from the Cambridge Structural Database (CSD). The remaining crystal structures are either publicly available as Supplementary Information from published papers, or can be requested from the authors of the respective databases presented in Table 2.
