# Supplementary material for: Exceptional hydrogen storage achieved by screening nearly half a million metal-organic frameworks
Source: Nat Commun. 2019 Apr 5;10:1568. doi: 10.1038/s41467-019-09365-w (PMC6450936; doi:10.1038/s41467-019-09365-w)
Supplement: Supplementary file 1 — Supplementary Information [file 41467_2019_9365_MOESM1_ESM.pdf]

Supplementary Information

Exceptional hydrogen storage achieved by screening nearly half a million metal-organic frameworks

Ahmed *et al.*

Supplementary Method

1. Computational details

1.1. Details of the MOF database

A database (DB) of 493,458 real and hypothetical MOFs was compiled, Table 1. The database includes 15,235 experimentally-derived MOF crystal structures from the UM[1], CoRE[2], and Cambridge Structural Database (CSD) 2017[3] databases. The UM and CoRE DBs are based on structures from the CSD versions 2011 and 2014 and were refined using an algorithm similar to that developed by Goldsmith *et al.* for solvent removal.[1,4] In addition to these 'real-MOF' databases, eight hypothetical MOF DBs from the literature were also examined.[5–10] Wilmer *et al.*[5] developed the first hypothetical database containing 137,000 MOFs constructed from 102 building blocks. Martin *et al.*[6] developed a DB of 116 MOF-5 analogs using commercially available ("Mail-Order") organic linkers. Bao *et al.*[7] used an evolutionary algorithm for the *de novo* design of 2,816 MOFs using an *in silico* technique for identifying appropriate linkers. Gomez-Gualdron *et al.*[8] designed a zirconium-based database consisting of 204 members using reverse topological engineering of 4 nets (fcu, ftw, scu, and csq). Finally, Aghaji *et al.*[9] generated a database of 320,000 hypothetical MOF structures combining 70 SBUs and 19 functional groups.

Supplementary Table 1. List of custom hypothetical or reconstructed MOFs examined in this work.

MOF Description	Density ($g\ cm^{-3}$)	Gravimetric Surface Area ($m^2\ g^{-1}$)	Volumetric Surface Area ($m^2\ cm^{-3}$)	Void Fraction	Pore Volume ($cm^3\ g^{-1}$)	Usable Gravimetric Capacity (wt.%)	Usable Volumetric Capacity ($g\ L^{-1}$)
Thiophenecarboxylateacrylate	0.46	4236	1961	0.85	1.84	7.0	36.8
Me-SNU-70	0.42	4569	1917	0.85	2.02	7.5	36.0
IRMOF-10_NIP	0.33	4999	1641	0.87	2.65	9.6	37.6
IRMOF-8_NIP	0.45	4379	1964	0.83	1.86	6.8	35.3
UMCM-8	0.51	4098	2096	0.82	1.61	5.7	33.4
UMCM-9	0.37	4847	1805	0.86	2.31	8.3	36.2
NU-110-anthracene	0.27	6000	1628	0.88	3.26	10.3	34.5
DichloroUMCM-1	0.42	4107	1709	0.85	2.04	6.9	33.7
DimethylUMCM-1	0.40	1713	4276	0.85	2.12	7.2	33.6
AnthraceneUMCM-1	0.43	3830	1640	0.84	1.95	6.3	31.7
dihydroisobenzofuranUMCM-1	0.41	4129	1677	0.85	2.09	7.1	33.9
NaphthaleneUMCM-1	0.41	4203	1719	0.85	2.07	6.9	33.2
Hydroxy-BPDC_IRMOF	0.36	4999	1809	0.87	2.39	8.6	36.4
Acetate-BPDC_IRMOF	0.45	5122	2281	0.83	1.85	6.3	32.3
NU-110-anthracene	0.27	6000	1628	0.88	3.26	10.3	34.5
BrMOF-5	1.3	1445	1911	0.76	0.57	1.5	21.0
MOF-5_25%_Ethynyl	0.61	3534	2154	0.80	1.31	3.6	25.4
MOF-177-NH_2	0.45	4514	2045	0.82	1.82	6.4	33.7

Supplementary Table 2. Examples of CSD 2017 MOFs not considered in the screening.[3]

MOF Identifier	Rationale for exclusion	CSD17 MOF	Rationale for exclusion	CSD17 MOF	Rationale for exclusion
VIJNOT	1D polymer	NATBAL	1D polymer	HACQOS	1D polymer
SUVMAY	1D polymer	ZODWAS	1D polymer	HACQUY	1D polymer
TEWMIS	1D polymer	HURHEI	2D polymer	HICLAH	1D polymer
NUSTIF	1D polymer	QIYKIU	1D polymer	LADQEM	1D polymer
PORZAZ	1D polymer	PEZVEW	1D polymer	LADQEM01	1D polymer
SIBDIS	1D polymer	HINQAY	1D polymer	NOZQEY	1D polymer
UZIDOX	1D polymer	KALZUU	1D polymer	TUBTOB	1D polymer
WUNPIE	1D polymer	BUQKOP	2D polymer	ULUBAE	1D polymer
HUBWUY	1D polymer	AXILOI	1D polymer	ULUBEI	1D polymer
LADQIQ	1D polymer	AHAZAM	2D polymer	WAJPED	2D polymer
SOFGIF	1D polymer	AFEJEB	2D polymer	XAWBOO	1D polymer
HICKOU	1D polymer	MEVBUK	1D polymer	XOTBUF	1D polymer
LADQOW	1D polymer	CERGIQ	1D polymer	COPBOZ	1D polymer
POBWIM	discrete complex	DAKYUL	1D polymer	WUYBIB	1D polymer
HUVBIK	1D polymer	DAKZEW	1D polymer	MAMKEQ	1D polymer
HUDHET	2D polymer	DIHKIP	1D polymer	YOQPEA	1D polymer
XOKYOM	1D polymer	EGUGIY	1D polymer	DAKXOE	1D polymer
KIQCIW	1D polymer	ENUKUU	1D polymer	ENAPAL	1D polymer
HIGMOA	1D polymer	GUTQIX	1D polymer	BIVVEI	1D polymer
BEWRUR	Doubly interpenetrated 3D MOF	BEMFOQ	1D polymer	FASZUW	1D polymer
BEWSAY	Doubly interpenetrated 3D MOF	AHAYUF	2D polymer	AHAYOZ	2D polymer
JOVKOW	2D polymer	GOVMEJ	2D polymer	FOXMIQ	1D polymer
ENIDOV	1D polymer	WOKLOZ	discrete metal complex	EGUGUK	1D polymer
GOBKEN	1D polymer	AHAYIT	1D polymer	EQADAC	1D polymer
BEJZIA	1D polymer	ICETER	1D polymer	BUKSAD	MOF, low experimental SA

1.2. Calculations of MOF's crystallographic properties

Single crystal density, pore volume, void fraction, pore diameter, gravimetric surface area, and volumetric surface areas of all MOFs were calculated using Zeo++ code[11] using a Voronoi decomposition method. Except for single crystal density, all other properties were computed using a N_2 probe molecule of radius 1.86 Å.

1.3. Details of Grand Canonical Monte Carlo simulations

A. Interatomic Potentials

Hydrogen molecules were modeled using the pseudo-Feynman-Hibbs model of Fischer et al.[12-14] This model has been extensively verified against measured hydrogen adsorption isotherms for MOF-5, IRMOF-20, UMCM-4, MOF-177, NH_2-MOF-177, and Cu-DUT-23, as reported in our earlier work.[14] MOF atoms were described using interatomic potential parameters from Refs. 15,16.

B. GCMC simulation conditions

All GCMC simulations were carried out using the RASPA[17] package. H_2-H_2 and H_2-MOF interactions were computed within a 12 Å cutoff radius. The MOF unit cell was replicated if smaller than 24 Å in length in any direction. Long range corrections were used to compensate for the exclusion of interactions beyond the cutoff radius.[18-19] All MOF structures were deemed to be rigid. Lorentz-Berthelot[20,21] combination rules were used in computing MOF-H_2 interaction parameters. Initially, H_2 capacity was determined at each pressure and temperature by averaging the number of H_2 molecules in the simulation cell over 1,000 GCMC production cycles, preceded by 1,000 initial cycles.[22] GCMC simulations were carried out using 20,000 cycles for a subset of high preforming MOFs; in these cases the last 10,000 cycles were used for computing H_2 adsorption. Each GCMC cycle was comprised of moves equal to the number of molecules in the system at the beginning of the cycle.[17] Translation, insertion, and deletion moves were performed with equal probabilities.[17] The average percentage deviations between short and long runs storage capacities are less than a few percent. That suggests short simulations are sufficient for high-throughput GCMC simulations of hydrogen uptakes, which is consistent with the conclusion recently drawn by Bobbit et al.[22]

1.4. MOF screening

Supplementary Figure S1 illustrates the workflow for computational screening. First, accessible surface areas and pore volumes were computed using the Zeo++ code. It was determined that 426,700 MOFs exhibit non-zero porosity or surface area. MOFs with zero accessible surface area were excluded from further screening. Second, the Chahine rule was used for computing total hydrogen storage capacities at 35 bar and 77 K as discussed in our earlier publications.[1,14] Third, MOFs that perform better than or equal to Chahine-rule-predicted MOF-5 capacities (i.e., 8.4 wt.% and 54.4 g L^{-1}) were retained for GCMC simulations. GCMC simulations were carried out on a total of 43,777 MOFs at 100 and 5 bar at 77 K. All MOFs contained in the real MOFs (UM+CoRE+CSD), mail-order, Zr-MOFs, and MOF-74 analogs databases and our custom-designed in-house MOFs as shown in Table 2 were screened using GCMC simulations without pre-screening. However, due to their large size, a multi-stage screening protocol as shown in Supplementary Figure S1 was used for screening rest of the hypothetical databases. Finally, MOFs were screened based on the usable capacity of MOF-5 (4.5 wt% & 31.1 g L^{-1}) and IRMOF-20 (5.7 wt.% & 33.4 g L^{-1}) for a pressure swing between 5 and 100 bar at 77 K.

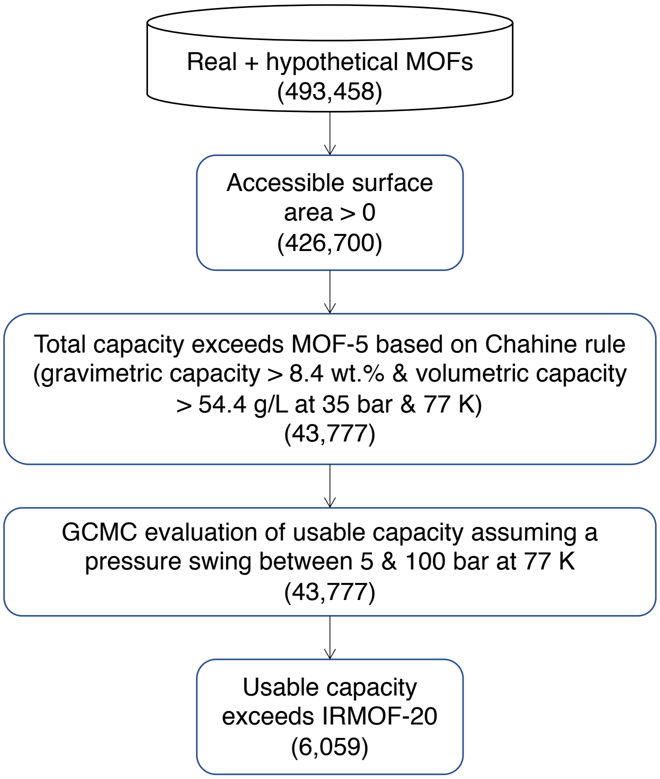

Supplementary Figure 1. Workflow for computational screening.

Supplementary Table 3. 50 Real MOFs that exceed the usable capacity of IRMOF-20.[1-3]

CSD Refcode	Single Crystal Density (g cm^{-3})	Gravimetric Surface Area (m^2 g^{-1})	Volumetric Surface Area (m^2 cm^{-3})	Void Fraction	Pore Volume (cm^3 g^{-1})	Largest Pore Diameter (Å)	Pore Limiting Diameter (Å)	Usable Gravimetric Capacity, Pressure Swing (wt.%)	Usable Volumetric Capacity, Pressure Swing (g L^{-1})
ECOLEP	0.41	4510	1836	0.89	2.09	11.6	10.9	8.2	39.0
VUSJUP	0.52	4142	2151	0.83	1.63	14.9	8.7	6.5	38.1
GAQYIH	0.56	3713	2079	0.84	1.52	20.3	9.0	6.1	37.8
XUKYEI	0.29	6327	1817	0.88	3.02	13.2	10.8	10.7	37.4
VEBHUG	0.45	4302	1936	0.87	1.89	17.3	10.0	7.2	37.4
BAZFUF	0.34	5470	1860	0.91	2.54	20.2	8.6	9.1	37.1
HABQUY	0.29	5750	1664	0.91	3.04	25.7	12.1	10.5	37.1
GAGZEV	0.28	5777	1613	0.92	3.17	28.7	11.5	10.8	37.0
ZELROZ	0.36	4947	1790	0.88	2.4	16.9	11.1	8.7	36.8
XAFFIV	0.36	5329	1910	0.89	2.36	14.2	13.2	8.5	36.6
VAGMAT	0.36	5203	1898	0.89	2.33	14.9	13.3	8.5	36.5
XAFFAN	0.37	5181	1892	0.89	2.33	14.9	13.2	8.3	36.5
NIBJAK	0.22	5417	1210	0.94	4.09	32.0	17.6	13.2	36.4
XAFFOB	0.37	5195	1907	0.89	2.32	14.8	13.2	8.3	36.4
HEXVEM	0.25	5455	1373	0.93	3.58	28.4	15.9	11.8	36.4
XAFFER	0.36	5171	1861	0.89	2.37	14.2	13.3	8.5	36.3
VAGMEX	0.35	5152	1815	0.9	2.43	15.3	14.5	8.7	36.3
NIBHOW	0.28	5103	1427	0.92	3.19	27.5	14.9	10.6	36.2
ADATIK	0.38	4566	1724	0.89	2.3	24.6	12.2	8.1	36.0
ADATAC	0.34	5145	1735	0.9	2.57	26.3	10.3	8.9	35.9
VETMIS	0.31	5713	1782	0.9	2.77	17.2	12.0	9.5	35.7
XAHPON	0.28	5268	1498	0.92	3.1	17.3	15.3	10.4	35.5
FEBXIV	0.29	5166	1517	0.91	3	17.3	15.8	10.1	35.5
LEJCIO	0.33	5275	1722	0.91	2.66	18.5	14.1	8.9	35.4
RUTNOK	0.24	6200	1493	0.9	3.73	24.6	14.7	12.1	35.4
MEHMET	0.41	4594	1878	0.89	2.06	21.8	9.1	7.3	35.2
LEJCEK	0.33	5776	1929	0.88	2.58	17.2	11.7	8.9	35.0
EHIJAH	0.39	4503	1734	0.88	2.21	18.5	11.7	7.6	35.0
EDUVOO	0.37	4857	1814	0.91	2.31	20.9	10.6	8.0	35.0
XAHPIH	0.36	4683	1668	0.89	2.42	14.3	13.4	8.2	35.0
HABRAF	0.38	4850	1854	0.89	2.21	24.3	9.0	7.8	35.0
LURRIA	0.41	4586	1864	0.92	2.08	22.4	9.7	7.2	34.9
XAHQAA	0.17	6250	1065	0.95	5.44	23.0	21.6	15.7	34.9
WIYMOG	0.41	6833	2788	0.81	2.05	12.1	7.6	7.3	34.8
XAFFUH	0.33	5152	1696	0.9	2.63	23.7	19.6	8.8	34.8
XAHPUT	0.18	6301	1126	0.94	5.15	21.8	20.6	14.9	34.7
ADASEF	0.44	4168	1816	0.89	1.96	21.6	10.9	6.8	34.5
HOMXIR	0.39	4388	1731	0.88	2.16	23.7	22.9	7.6	34.5
ECOKAJ	0.33	3575	1163	0.89	2.69	19.0	17.6	8.9	34.5
BAZGAM	0.13	6581	833	0.97	7.46	42.8	24.2	19.3	34.3
BIBXOB	0.41	4924	2017	0.87	2.04	19.7	8.0	7.2	34.2
HOHMEX	0.32	4986	1575	0.88	2.74	18.8	14.9	9.0	34.1
PIBPIA	0.46	2982	1368	0.85	1.83	15.5	14.3	6.6	34.1
XAHPED	0.37	5131	1921	0.87	2.26	12.4	10.9	7.8	34.0
PIBNUK	0.42	3289	1391	0.85	1.98	15.4	14.2	7.1	34.0
ALULEZ	0.43	3447	1468	0.84	1.96	18.8	13.4	6.9	34.0
DITJIB	0.52	3398	1772	0.87	1.6	20.4	9.0	5.8	33.9
RICBEM	0.4	5745	2293	0.88	2.07	11.4	8.6	7.1	33.9
LEHXUT	0.41	4560	1857	0.88	2.06	25.0	9.1	7.1	33.9
PIBNUK01	0.42	3297	1394	0.85	1.97	15.4	14.2	7.0	33.8

Supplementary Table 4. Mail-order MOFs that exceed the usable capacity of IRMOF-20.[23]

MOF Name	Single Crystal Density (g cm^{-3})	Gravimetric Surface Area (m^2 g^{-1})	Volumetric Surface Area (m^2 cm^{-3})	Void Fraction	Pore Volume (cm^3 g^{-1})	Largest Pore Diameter (Å)	Pore Limiting Diameter (Å)	Usable Gravimetric Capacity, Pressure Swing (wt.%)	Usable Volumetric Capacity, Pressure Swing (g L^{-1})
MOF-5_cooh_2_2738_1_basic_opt	0.47	4548	2149	0.78	1.34	7.8	15.8	7.1	39.3
MOF-5_cooh_2_2796_1_basic_opt	0.37	4965	1838	0.87	2.36	10.0	16.4	8.8	37.8
MOF-5_cooh_2_394_1_basic_opt	0.29	5743	1640	0.89	3.13	11.8	20.3	10.9	36.9
MOF-5_cooh_2_68_1_basic_opt	0.32	5233	1679	0.88	2.74	11.1	20.1	9.7	36.9
MOF-5_cooh_2_567_1_basic_opt	0.40	4756	1905	0.86	2.14	10.0	16.5	8.0	36.8
MOF-5_cooh_2_2368_1_basic_opt	0.23	5938	1351	0.91	4.01	14.7	23.4	13.1	36.7
MOF-5_cooh_2_646_1_basic_opt	0.24	5781	1392	0.91	3.76	14.0	22.3	12.5	36.7
MOF-5_cooh_2_790_1_basic_opt	0.30	5149	1529	0.89	2.99	13.0	21.6	10.3	36.6
MOF-5_cooh_2_1929_1_basic_opt	0.45	4045	1823	0.84	1.87	9.5	17.8	7.0	36.5
MOF-5_cooh_2_1505_1_basic_opt	0.25	5714	1421	0.91	3.64	13.6	22.3	12.1	36.4
MOF-5_cooh_2_239_2_basic_opt	0.49	4225	2071	0.84	1.72	8.7	13.7	6.6	36.4
MOF-5_cooh_2_1861_1_basic_opt	0.30	5236	1594	0.88	2.90	12.1	20.3	10.1	36.3
MOF-5_cooh_2_11_1_basic_opt	0.33	5282	1746	0.87	2.65	12.3	18.1	9.3	36.0
MOF-5_cooh_2_2349_1_basic_opt	0.26	5948	1548	0.90	3.45	12.6	21.2	11.4	35.9
MOF-5_cooh_2_2558_1_basic_opt	0.21	5955	1262	0.91	4.31	15.1	24.3	13.5	35.8
MOF-5_cooh_2_1239_1_basic_opt	0.29	5834	1699	0.88	3.03	11.3	19.8	10.2	35.7
MOF-5_cooh_2_861_1_basic_opt	0.42	4556	1929	0.84	1.98	9.3	16.2	7.3	35.3
MOF-5_cooh_2_779_1_basic_opt	0.13	6997	934	0.94	7.07	20.4	30.9	19.1	34.3
MOF-5_cooh_2_1589_1_basic_opt	0.14	6581	940	0.94	6.59	20.7	31.3	18.1	34.1

Supplementary Table 5. *In silico* deliverable MOFs that exceed the usable capacity of IRMOF-20.[7]

MOF Name	Single Crystal Density (g cm^{-3})	Grav. Surface Area (m^2 g^{-1})	Vol. Surface Area (m^2 cm^{-3})	Void Fraction	Pore Volume (cm^3 g^{-1})	Largest Pore Diameter (Å)	Pore Limiting Diameter (Å)	Usable Grav. Capacity, Pressure Swing (wt.%)	Usable Volumetric Capacity, Pressure Swing (g L^{-1})
Syn014648	0.48	4686	2248	0.84	1.75	11.1	7.8	7.0	38.2
Syn028362	0.40	5733	2272	0.83	2.10	11.7	9.2	7.6	35.3
Syn031169	0.47	4833	2294	0.83	1.75	11.4	8.5	6.5	34.9
Syn029009	0.40	5449	2204	0.82	2.04	12.0	9.2	7.4	34.6
Syn015166	0.42	5329	2240	0.83	1.97	11.4	8.8	7.0	34.2
Syn014460	0.50	4310	2172	0.83	1.64	16.3	8.8	5.9	33.6

Supplementary Table 6. *In silico* surface MOFs that exceed the usable capacity of IRMOF-20.[10]

MOF Name	Single Crystal Density (g cm^{-3})	Grav. Surface Area (m^2 g^{-1})	Vol. Surface Area (m^2 cm^{-3})	Void Frac.	Pore Vol. (cm^3 g^{-1})	Largest Pore Diameter (Å)	Pore Limiting Diameter (Å)	Usable Grav. Capacity, Pressure Swing (wt.%)	Usable Vol. Capacity, Pressure Swing (g L^{-1})
cds_Syn029752	0.45	4898	2192	0.83	1.86	2.6	11.9	7.2	36.9
cds_Syn027014	0.40	5484	2191	0.84	2.11	3.0	12.2	7.9	36.7
cds_Syn015279	0.43	5075	2179	0.84	1.97	3.2	14.0	7.4	36.7
cds_Syn034835	0.42	5346	2262	0.84	1.97	2.8	12.4	7.5	36.6
cds_Syn025813	0.42	5218	2210	0.85	2.00	3.2	13.9	7.4	36.4
cds_Syn032331	0.43	5170	2204	0.84	1.97	2.8	12.4	7.4	36.3
cds_Syn035762	0.42	5287	2213	0.84	2.01	3.3	14.3	7.5	36.3
sod_B_Syn000038	0.38	5836	2232	0.84	2.20	3.3	12.9	8.1	36.0
cds_Syn038557	0.48	4740	2294	0.83	1.72	3.0	14.8	6.5	35.9
cds_Syn025253	0.43	5108	2206	0.84	1.94	3.2	14.5	7.2	35.9
cds_Syn024908	0.46	4900	2241	0.83	1.82	2.8	13.4	6.9	35.9
cds_A_Syn008586	0.38	5938	2254	0.84	2.22	3.6	14.3	8.0	35.8
cds_Syn037641	0.46	4990	2271	0.83	1.83	3.3	15.4	6.8	35.5
cds_Syn035184	0.44	5085	2251	0.83	1.88	3.2	14.4	7.0	35.4
cds_Syn024117	0.45	4982	2221	0.84	1.87	3.3	15.2	6.9	35.3
cds_Syn030154	0.44	5307	2322	0.83	1.90	3.3	14.8	7.0	35.3
cds_Syn039995	0.43	5203	2237	0.84	1.95	3.5	15.5	7.1	35.2
sod_B_Syn000903	0.37	5956	2216	0.83	2.24	3.7	14.4	8.0	35.2
cds_Syn024859	0.45	4995	2261	0.83	1.84	3.3	15.4	6.8	35.1
cds_Syn030819	0.49	4767	2340	0.83	1.68	3.0	15.3	6.3	35.1

Supplementary Table 7. ToBaCCo MOFs that exceed the usable capacity of IRMOF-20.[24]

MOF Name	Single Crystal Density (g cm^{-3})	Grav. Surface Area (m^2 g^{-1})	Vol. Surface Area (m^2 cm^{-3})	Void Frac.	Pore Vol. (cm^3 g^{-1})	Largest Pore Diameter (Å)	Pore Limiting Diameter (Å)	Usable Grav. Capacity, Pressure Swing (wt.%)	Usable Vol. Capacity, Pressure Swing (g L^{-1})
mof_4690	0.33	7327	2437	0.86	2.59	12.4	12.2	9.7	38.7
mof_7599	0.38	5589	2127	0.85	2.24	12.7	9.0	8.5	38.1
mof_4699	0.35	6949	2461	0.86	2.42	13.4	13.1	9.0	37.8
mof_4639	0.38	5876	2246	0.85	2.22	13.3	11.3	8.4	37.8
mof_6830	0.40	5404	2139	0.84	2.13	16.5	7.7	8.2	37.6
mof_4707	0.36	6546	2359	0.86	2.38	14.8	14.3	8.8	37.6
mof_6831	0.38	5664	2177	0.85	2.20	15.8	8.4	8.4	37.5
mof_4738	0.36	6848	2447	0.85	2.38	12.4	12.2	8.8	37.5
mof_4978	0.36	6815	2439	0.85	2.38	12.4	12.2	8.8	37.4
mof_4930	0.34	7160	2469	0.85	2.48	12.9	11.6	9.1	37.4
mof_4947	0.36	6572	2378	0.86	2.37	14.8	14.0	8.7	37.4
mof_4952	0.27	8067	2216	0.87	3.17	15.8	15.1	11.0	37.3
mof_4939	0.36	6968	2496	0.86	2.39	12.7	12.6	8.7	37.3
mof_6954	0.44	5044	2229	0.84	1.90	16.2	7.2	7.3	37.1
mof_4747	0.37	6461	2419	0.85	2.27	13.6	13.2	8.3	36.8
mof_6522	0.43	4922	2140	0.84	1.93	10.4	9.3	7.3	36.7
mof_4987	0.38	6401	2414	0.85	2.25	13.6	13.2	8.2	36.6
mof_6074	0.43	4946	2132	0.84	1.96	11.9	9.5	7.4	36.5
mof_3988	0.32	6732	2185	0.84	2.58	12.7	9.0	9.3	36.3
mof_4995	0.37	6154	2305	0.85	2.28	15.3	15.0	8.2	36.3

Supplementary Table 8. Top ranked Zr-MOFs that exceed the usable capacity of IRMOF-20.[8]

MOF Name	Single Crystal Density (g cm^{-3})	Grav. Surface Area (m^2 g^{-1})	Vol. Surface Area (m^2 cm^{-3})	Void Frac.	Pore Vol. (cm^3 g^{-1})	Largest Pore Diameter (Å)	Pore Limiting Diameter (Å)	Usable Grav. Capacity, Pressure Swing (wt.%)	Usable Vol. Capacity, Pressure Swing (g L^{-1})
NU-TPE-4PTT-ftw	0.27	6323	1684	0.88	3.30	10.8	21.6	11.5	37.5
NU-Pyr-4PTT-ftw	0.33	5741	1875	0.86	2.64	10.2	21.4	9.5	37.3
NU-Por-4PTT-ftw	0.33	5576	1836	0.86	2.61	8.9	22.0	9.4	37.2
NU-TPE-4TTP	0.27	5838	1569	0.88	3.27	11.2	22.4	11.4	37.0
NU-TPE-4TPT-ftw	0.27	6335	1704	0.88	3.26	11.1	22.5	11.2	36.9
NU-Pyr-4TTP-ftw	0.33	5144	1678	0.86	2.63	10.6	19.4	9.5	36.8
NU-Py-4PTT-scu-s	0.28	5438	1531	0.89	3.15	18.0	20.1	10.9	36.8
NU-2_P_4PTT_Por_PTT-ftw	0.37	5469	2002	0.84	2.31	9.3	16.8	8.5	36.7
NU-P-4TTP-scu-s	0.35	4774	1655	0.86	2.49	13.4	17.9	9.1	36.7
NU-Por-4TTP-ftw	0.32	5209	1672	0.86	2.68	9.7	22.3	9.6	36.7
NU-P-4PTT-scu-s	0.35	4988	1728	0.86	2.50	13.8	15.9	9.0	36.6
NU-TPE-4TPT-scu-s	0.28	5450	1517	0.88	3.17	15.2	19.0	11.0	36.6
NU-TPE-4PTT-scu-s	0.28	5703	1587	0.88	3.17	15.8	18.4	11.0	36.6
NU-Por-4PTT-scu	0.26	5461	1446	0.89	3.37	19.0	20.5	11.5	36.6
NU-Py-4TPT-scu-s	0.28	5407	1512	0.89	3.17	18.2	22.5	10.8	36.5
NU-P-4TPT-scu-s	0.35	4903	1700	0.86	2.49	13.2	18.2	9.0	36.5
NU-TTTT-fcu	0.48	4262	2041	0.83	1.74	8.6	17.3	6.7	36.5
NU-TPE-4PTT-scu-l	0.24	5863	1383	0.90	3.82	18.4	21.1	12.6	36.5
NU-TPE-4TPT-scu-l	0.24	5754	1356	0.90	3.82	18.1	21.4	12.6	36.4
NU-Py-4TTP-scu-s	0.27	5126	1404	0.89	3.25	17.7	21.1	10.9	36.4

Supplementary Table 9. Top ranked Northwestern MOFs that exceed the usable capacity of IRMOF-20.[5]

MOF Name	Single Crystal Density (g cm^{-3})	Grav. Surface Area ($m^2 g^{-1}$)	Vol. Surface Area ($m^2 cm^{-3}$)	Void Frac.	Pore Vol. ($cm^3 g^{-1}$)	Largest Pore Diameter (Å)	Pore Limiting Diameter (Å)	Usable Grav. Capacity, Pressure Swing (wt.%)	Usable Vol. Capacity, Pressure Swing (g L^{-1})
hypotheticalMOF_5048108_i_1_j_25_k_20_m_3	0.40	5285	2140	0.86	2.12	10.3	12.5	8.2	38.1
hypotheticalMOF_5048221_i_1_j_25_k_20_m_10	0.35	6165	2144	0.86	2.47	10.3	12.6	9.3	38.1
hypotheticalMOF_3000771_i_1_j_26_k_24_m_0_cat_1	0.40	5762	2333	0.85	2.11	8.1	10.9	8.2	37.9
hypotheticalMOF_5072982_i_2_j_25_k_20_m_2	0.37	5758	2144	0.86	2.30	9.7	12.7	8.7	37.9
hypotheticalMOF_5018670_i_0_j_25_k_19_m_11	0.42	5124	2143	0.85	2.04	10.6	12.8	7.9	37.9
hypotheticalMOF_5048082_i_1_j_25_k_20_m_1	0.37	5808	2143	0.86	2.33	10.3	13.2	8.8	37.9
hypotheticalMOF_5073022_i_2_j_25_k_20_m_4	0.35	6114	2137	0.86	2.45	9.7	12.4	9.2	37.9
hypotheticalMOF_3000644_i_1_j_26_k_23_m_0_cat_1	0.41	5831	2382	0.85	2.08	8.1	10.6	8.1	37.8
hypotheticalMOF_5038380_i_1_j_20_k_19_m_14	0.43	4962	2133	0.84	1.96	8.4	12.6	7.7	37.8
hypotheticalMOF_5072986_i_2_j_25_k_20_m_3	0.42	5200	2192	0.85	2.02	9.7	12.3	7.8	37.8
hypotheticalMOF_5048278_i_1_j_25_k_21_m_0	0.35	6163	2134	0.86	2.48	9.7	12.7	9.3	37.8
hypotheticalMOF_5001093_i_0_j_19_k_6_m_13	0.40	5342	2157	0.85	2.11	9.0	12.1	8.1	37.8
hypotheticalMOF_5072970_i_2_j_25_k_20_m_2	0.37	5725	2131	0.86	2.30	9.7	12.4	8.7	37.8
hypotheticalMOF_5018606_i_0_j_25_k_19_m_6	0.42	5222	2169	0.85	2.05	10.6	12.3	7.9	37.7
hypotheticalMOF_5018699_i_0_j_25_k_19_m_14	0.42	5142	2136	0.86	2.07	10.6	13.4	7.9	37.7
hypotheticalMOF_5072946_i_2_j_25_k_20_m_1	0.36	5843	2127	0.86	2.35	9.7	12.8	8.9	37.7
hypotheticalMOF_5072954_i_2_j_25_k_20_m_1	0.36	5871	2137	0.86	2.36	9.7	13.2	8.9	37.6
hypotheticalMOF_5039680_i_1_j_21_k_11_m_1	0.38	5676	2153	0.86	2.26	8.8	13.2	8.6	37.6
hypotheticalMOF_5053154_i_1_j_27_k_21_m_11	0.36	5877	2138	0.86	2.35	9.7	12.3	8.8	37.6
hypotheticalMOF_5041161_i_1_j_21_k_21_m_14	0.36	5972	2165	0.86	2.36	9.2	13.4	8.9	37.6

Supplementary Table 10. Top ranked University of Ottawa MOFs that exceed the usable capacity of IRMOF-20.[9]

MOF Name	Single Crystal Density (g cm^{-3})	Grav. Surface Area (m^2 g^{-1})	Vol. Surface Area (m^2 cm^{-3})	Void Frac.	Pore Vol. (cm^3 g^{-1})	Largest Pore Diameter (Å)	Pore Limiting Diameter (Å)	Usable Grav. Capacity, Pressure Swing (wt.%)	Usable Vol. Capacity, Pressure Swing (g L^{-1})
str_m3_o5_o25_f0_nbo.sym.193.out	0.42	5147	2166	0.86	2.04	13.3	7.9	7.9	38.2
str_m2_o5_o25_f0_nbo.sym.167.out	0.42	5119	2142	0.86	2.05	13.2	7.9	7.9	38.2
str_m3_o20_o21_f0_pcu.sym.19.out	0.40	5428	2151	0.85	2.16	12.8	10.3	8.3	38.2
str_m2_o20_o25_f0_pcu.sym.10.out	0.36	5957	2170	0.86	2.36	12.5	9.8	8.9	38.1
str_m3_o5_o25_f0_nbo.sym.19.out	0.43	5031	2179	0.86	1.98	13.3	8.3	7.7	38.1
str_m2_o5_o28_f0_nbo.sym.24.out	0.41	5164	2132	0.85	2.06	12.9	7.2	8.0	38.1
str_m2_o5_o25_f0_nbo.sym.110.out	0.41	5255	2156	0.86	2.09	13.3	7.9	8.1	38.0
str_m2_o5_o25_f0_nbo.sym.112.out	0.43	5081	2178	0.85	1.99	13.2	7.9	7.8	38.0
str_m2_o20_o25_f0_pcu.sym.33.out	0.37	5817	2139	0.86	2.34	13.2	9.8	8.8	38.0
str_m2_o5_o25_f0_nbo.sym.115.out	0.43	5030	2154	0.85	1.99	13.2	7.9	7.8	38.0
str_m2_o5_o25_f0_nbo.sym.35.out	0.42	5147	2174	0.86	2.03	13.4	7.9	7.8	38.0
str_m2_o5_o25_f0_nbo.sym.122.out	0.41	5319	2180	0.86	2.09	13.3	7.9	8.1	38.0
str_m3_o5_o25_f0_nbo.sym.139.out	0.43	5049	2183	0.86	1.98	13.3	7.8	7.6	38.0
str_m2_o20_o25_f0_pcu.sym.31.out	0.36	6037	2157	0.86	2.40	12.9	9.8	9.1	38.0
str_m2_o5_o25_f0_nbo.sym.132.out	0.42	5127	2166	0.86	2.03	13.2	8.3	7.8	37.9
str_m2_o20_o25_f0_pcu.sym.23.out	0.36	5918	2133	0.86	2.38	13.2	9.8	9.0	37.9
str_m3_o5_o25_f0_nbo.sym.173.out	0.46	4780	2199	0.85	1.86	12.8	8.0	7.2	37.9
str_m2_o20_o25_f0_pcu.sym.67.out	0.36	5951	2168	0.86	2.36	12.5	9.8	8.9	37.9
str_m3_o20_o25_f0_pcu.sym.28.out	0.42	5196	2178	0.86	2.05	13.0	10.1	7.8	37.9
str_m3_o5_o25_f0_nbo.sym.44.out	0.43	5103	2197	0.86	1.99	13.3	8.3	7.7	37.9

2. Measured nitrogen adsorption isotherms used in BET surface estimations

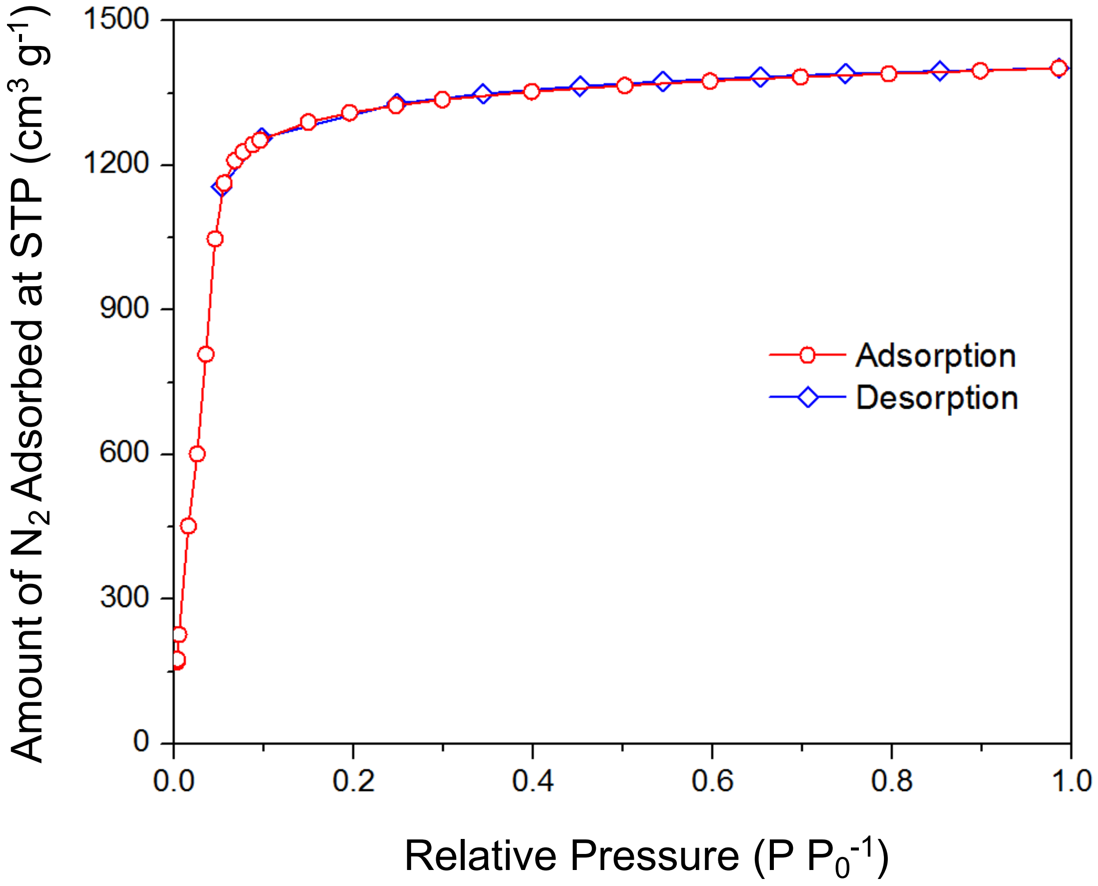

Supplementary Figure 2. N_2 Isotherm of UMCM-9.

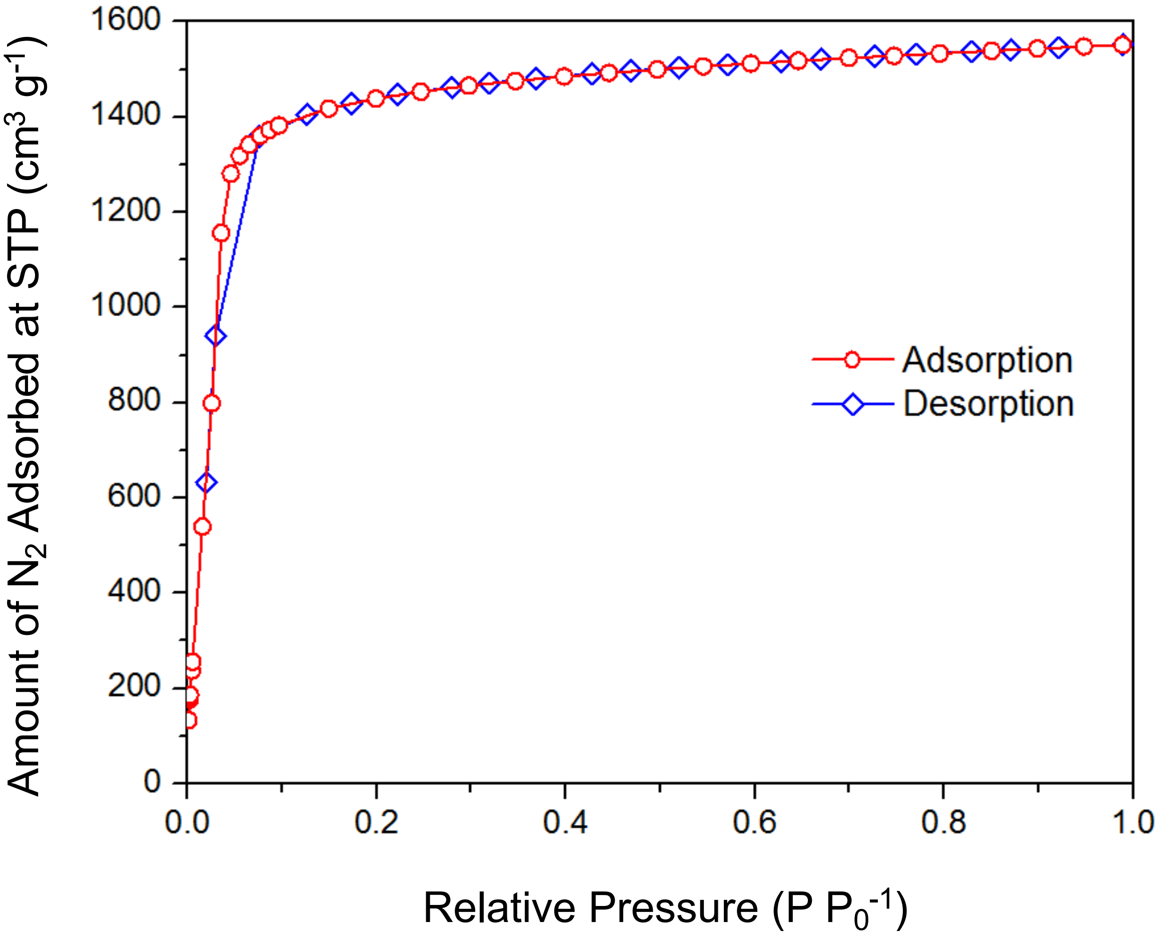

Supplementary Figure 3. N_2 Isotherm of SNU-70.

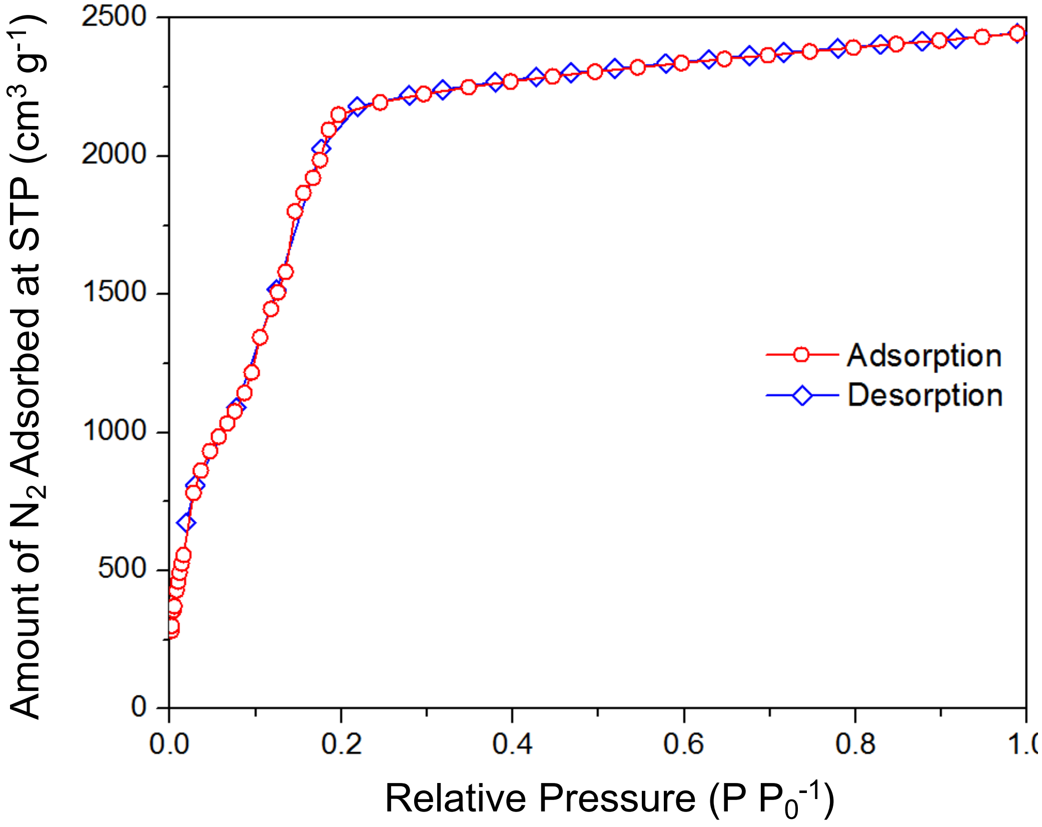

Supplementary Figure 4. N_2 Isotherm of NU-100/PCN-610.

3. Comparison between measured and GCMC calculated H_2 adsorption isotherms

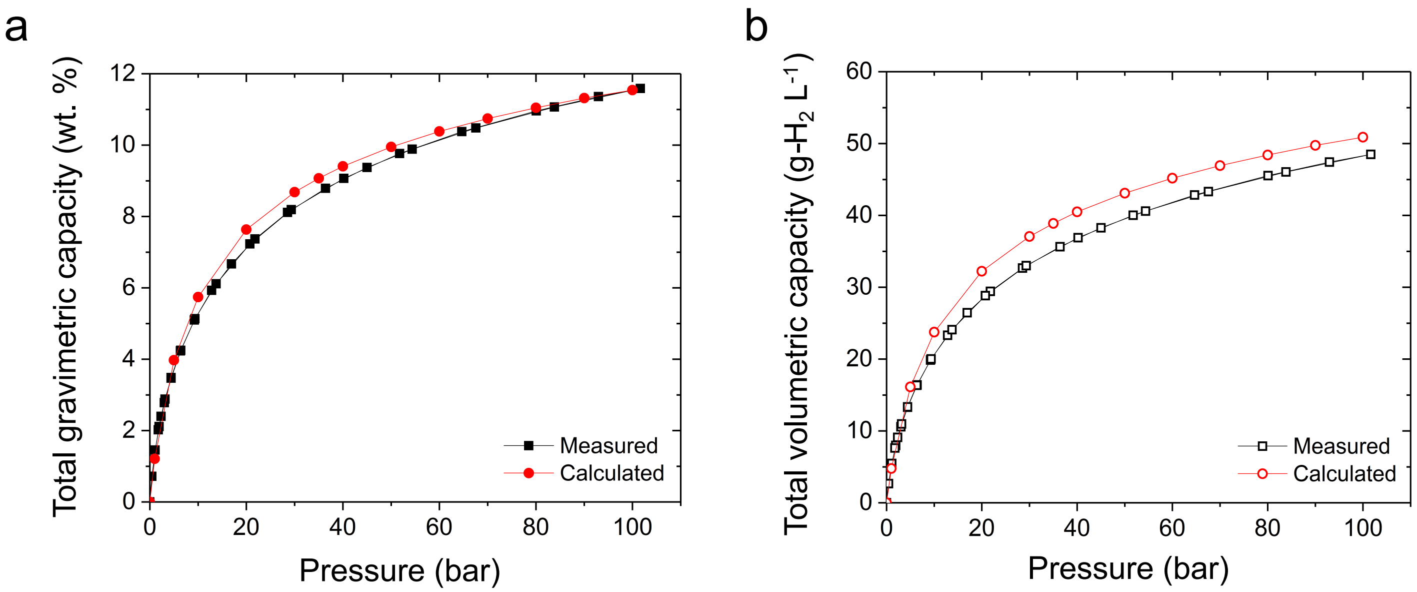

Supplementary Figure 5. Comparison between measured and GCMC calculated total (a) gravimetric and (b) volumetric H_2 adsorption isotherms of UMCM-9 at 77 K.

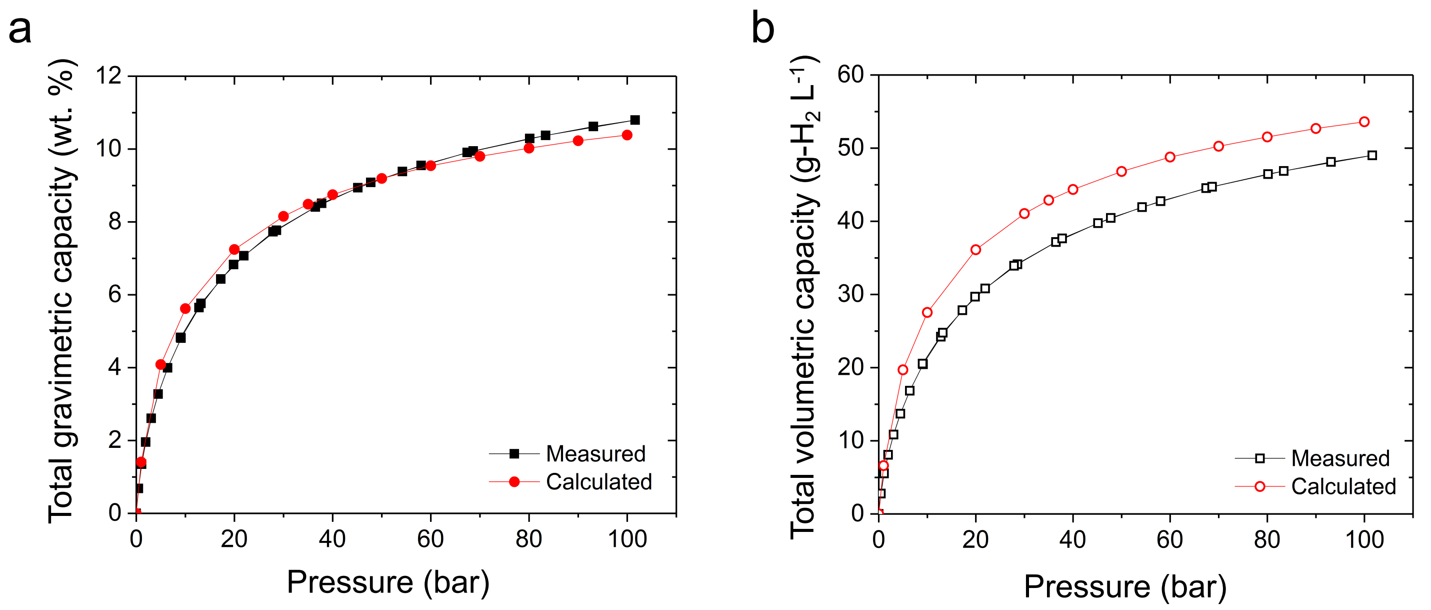

Supplementary Figure 6. Comparison between measured and GCMC calculated total (a) gravimetric and (b) volumetric H_2 adsorption isotherms of SNU-70 at 77 K.

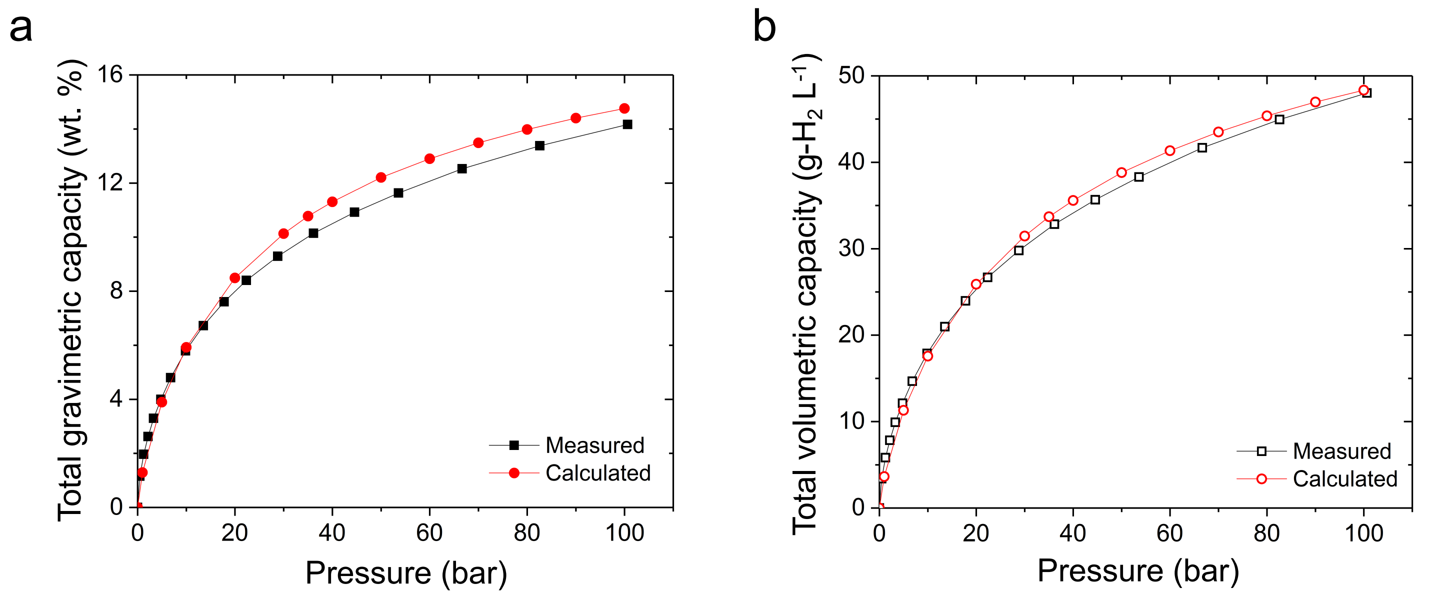

Supplementary Figure 7. Comparison between measured and GCMC calculated total (a) gravimetric and (b) volumetric H_2 adsorption isotherms of NU-100/PCN-610 at 77 K.

4. Experimental details
4.1. General considerations

All reagents were obtained from commercial sources and used without further purification unless otherwise mentioned. Phase purity of the MOFs was determined prior to activation by powder X-ray diffraction (PXRD) on a Rigaku Smartlab diffractometer using Cu-Kα radiation (λ = 1.54187 Å) operating at 40 kV and 44 mA. The MOFs soaked in DMF were packed in a glass capillary, and PXRDs were recorded in transmission mode using a point focus source (0.5 mm collimator) and a 2D Pilatus detector. The powder diffraction patterns were in good agreement with their respective powder patterns simulated from the single crystal structures. BET surface areas and pore volumes of the MOFs were calculated from the nitrogen adsorption and desorption isotherm at 77 K from 0.005 to 1 bar using a NOVA e-series 4200 surface area analyzer from Quantachrome Instruments (Boynton Beach, Florida, USA).

4.2. MOF synthesis and activation procedure
4.2.1 Synthesis and activation of PCN-610/NU-100
A. Ligand synthesis scheme for PCN-610/NU-100

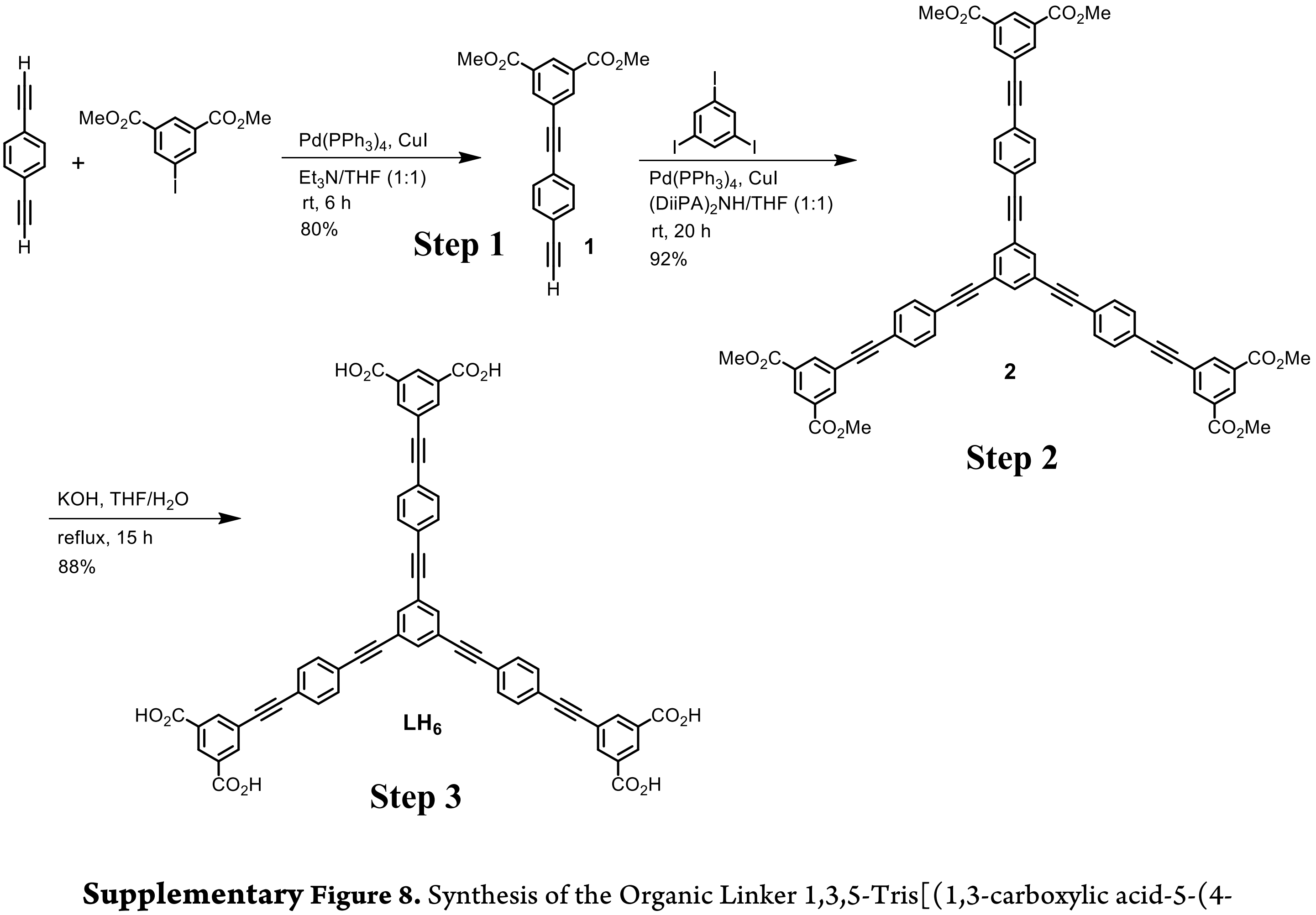

Supplementary Figure 8. Synthesis of the Organic Linker 1,3,5-Tris[(1,3-carboxylic acid-5-(4-(ethynyl)phenyl))ethynyl] benzene (LH_6).

Step-1: *Dimethyl 5-((4-ethynylphenyl)ethynyl)isophthalate* (**1**)

In a 250 mL round bottom flask were added tetrahydrofuran (THF, 60 mL) and triethylamine (Et_3N, 60 mL), and nitrogen was bubbled through the solution for 15 min. To the solution were added 1,4-diethynylbenzene (1.575 g, 12.5 mmol), methyl 3-iodoisophthalate (1.000 g, 3.125 mmol), tetrakis(triphenylphosphine)palladium (0.060 g, 0.052 mmol) and cuprous iodide (0.010 g, 0.0525 mmol) under nitrogen atmosphere, and the resulting mixture was stirred under nitrogen at room temperature. The progress of the reaction was monitored by TLC analysis. After about 8 h the iodoester was consumed as observed in TLC. The reaction mixture was filtered through celite, and the residue was washed with 20 mL of 1:1 THF/Et_3N mixture, followed by 15 mL chloroform. The combined organic layer was evaporated to obtain the crude product. The crude material was purified by column chromatography on silica gel to obtain the pure product as off white solid (0.796 g, 80%).

Step-2: *1,3,5-Tris[(1,3-dimethylcarboxylate-5-(4-(ethynyl)phenyl))ethynyl]benzene* (**2**)

A mixture of THF (80 mL) and diisopropylamine (60 mL) was taken in a 250 mL round bottom flask, and nitrogen was bubbled through the solution for 15 min. To the solution were added 1,3,5-triiodobenzene (0.501 g, 1.099 mmol), compound **1** (1.4 g, 4.398 mmol), tetrakis(triphenylphosphine)palladium (0.063 g, 0.055 mmol) and cuprous iodide (0.010 g, 0.0525 mmol). The mixture was stirred at room temperature for 3h. The reaction mixture was filtered, and residual solid washed with 10 mL THF to obtain the crude product. The crude product was dispersed in THF, stirred for 15 min, and then filtered to obtain the pure product as pale yellow solid (1.039 g, 92%).

Step-3: *1,3,5-Tris[(1,3-carboxylic acid-5-(4-(ethynyl)phenyl))ethynyl]benzene* (**LH_6**)

To the compound **2** (1.008 g, 0.981 mmol) taken in a round bottom flask was added 40 mL THF. KOH (2.006 g, 35.821 mmol) was dissolved in 40 mL water, the solution was slowly added to the THF solution of the ester, and the resulting mixture was refluxed for 15 h. The reaction mixture was then cooled down to room temperature, THF was removed in vacuuo, and the remaining solution was acidified by addition of c. HCl. The product was collected by centrifugation, washed with deionized water, and dried under vacuuo to obtain the pure product (0.814 g, 88 %).

B. Synthesis and activation of PCN-610/NU-100

NU-100 was synthesized following the literature procedure.[25] 1,3,5-Tris[(1,3-carboxylic acid-5-(4-(ethynyl)phenyl)) ethynyl]benzene (LH_6) (0.300 g, 0.32 mmol) and $Cu(NO_3)_2{\cdot}2.5H_2O$ (0.600 g, 2.579 mmol) were dissolved in 36 mL DMF in a glass vial. Subsequently, 0.2 mL HBF_4 was added to the solution, and the color of the solution turned teal. The solution was divided into thirty 4 mL vials (1.2 mL solution in each vial), and the vials were heated to 75 °C for 20 h. Teal colored octahedral crystals were formed at the bottom of the vial, which were collected together in a 60 mL jar, immersed in DMF for one day, and the supernatant liquid was replaced with fresh DMF (20 mL×4) in this time. Subsequently, the MOF was immersed in ethanol for another day, and the liquid was replaced with fresh ethanol four times (20 mL×4). The compound was then activated by flowing liquid CO_2 at 2 mL min^{-1} flowrate for 1 h at room temperature, subsequently by supercritical CO_2 at 2 mL min^{-1} flowrate for 2 h at 55 °C, and finally by supercritical CO_2 at 1 mL min^{-1} flowrate for 6 h at 55 °C to result a purple solid (0.123 g, 34.4 % based on LH_6).

4.2.2 Synthesis and activation of UMCM-9

UMCM-9 was synthesized following the reported procedure.[26] In five 60 mL glass jars with teflon-lined lids were added naphthalene-2,6-dicarboxylic acid (H_2NDC, 0.0285 mg, 0.131 mmol), 1,1′-biphenyl-4,4′-dicarboxylic acid (H_2BPDC, 0.0354 mg, 0.146 mmol), 6.7 mL of DEF and 13.3 mL of N-methylpyrrolidone, and the solids were dissolved in the solvent mixtures by sonication. Subsequently, $Zn(NO_3)_2 \cdot 6H_2O$ (0.235 g, 0.790 mmol) was added to the solution and the mixture was sonicated until a transparent solutions were obtained. The reaction mixtures were heated to 85 °C for 4 days. Cubic crystals of UMCM-9 were formed at the inner surface of the vials along with minor amount of flocculent precipitate. After cooling to room temperature the mother liquor was decanted, the precipitate was removed by multiple DMF washes, and the crystals were collected together in a different vial. The MOF crystals were immersed in DMF for 3 days (washed several times with fresh DMF), then in dichloromethane for 18 hours (washed with DCM, 20 mL×8), and finally, in dry *n*-hexane for 12 hours (washed with dry *n*-hexane 20 mL×4). Subsequently, the solvent was decanted, the vial was placed in a vacuum chamber, and exposed to vacuum very slowly at room temperature. Finally, the material was activated under high vacuum (below 10^{-4} torr) for 26 hours to yield clear pale yellow crystals (average yield 0.0523 g, 38%, based on H_2NDC).

4.2.3 Synthesis and activation of SNU-70

SNU-70 was synthesized following the reported procedure with slight modification.[27] (E)-4-(2-Carboxyvinyl)benzoic acid (0.075 g, 0.390 mmol) and $Zn(NO_3)_2 \cdot 6H_2O$ (0.150 g, 0.504 mmol) were dissolved in 25 mL DEF in a 60 mL glass jars with a teflon-lined lid. Six such reaction mixtures were heated to 105 °C for 12.5 h. At the end of this period, the glass jars were removed from the oven, and allowed to cool down to room temperature. Colorless cubic crystals (along with some fluffy precipitate) were formed at the bottom and the wall of the jars. The fluffy precipitate was removed from the MOF crystals by multiple wash with DMF. The remaining crystals were then collected together in a 60 mL glass vial, soaked in DMF and kept emerged for 2 d. The supernatant liquid was replaced with fresh DMF six times (20 mL each) in this time. The material was activated by SC CO_2 flow by the same procedure as NU-100 (0.567 g, 51%).

4.3. NU-100 ligand characterization via NMR spectroscopy

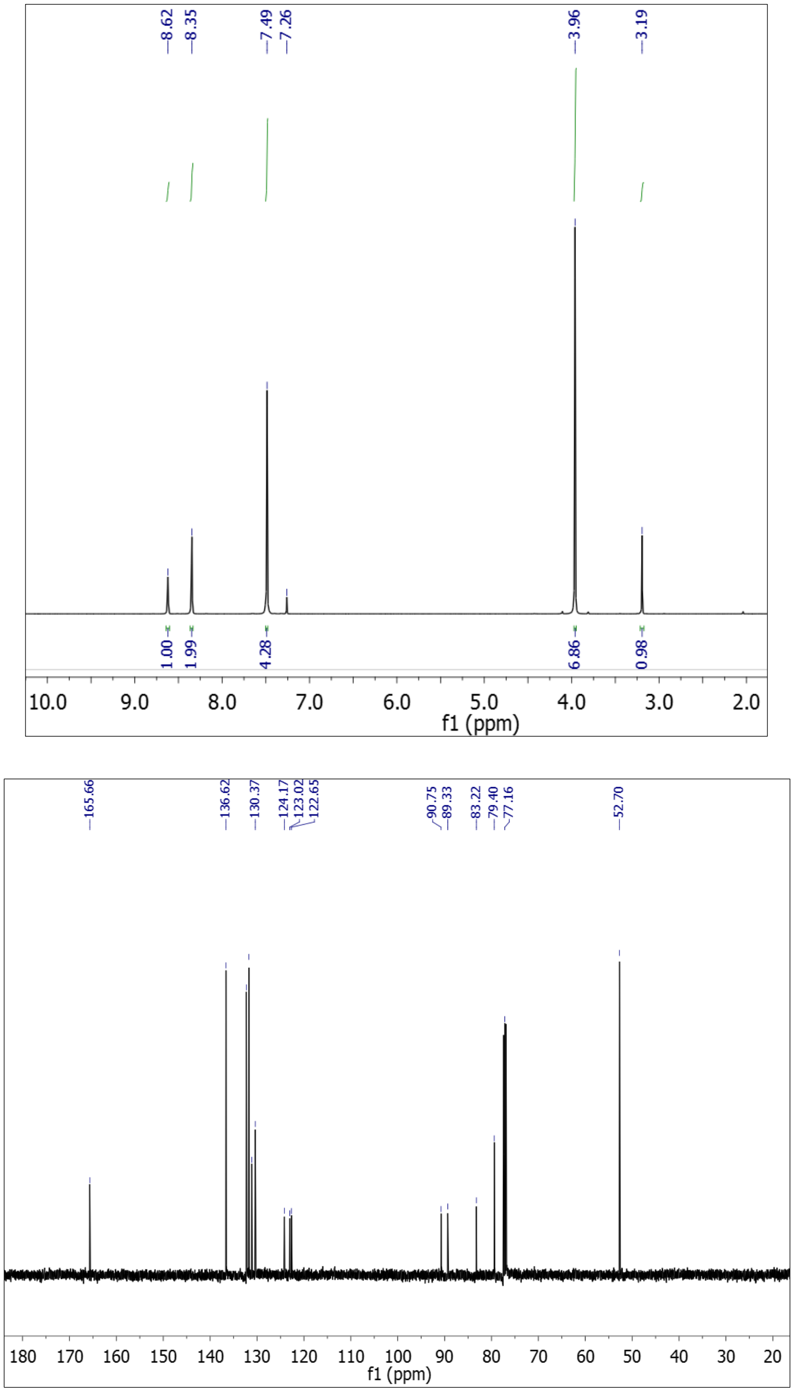

Supplementary Figure 9. ^1H (500 MHz) and ^{13}C (125 MHz) NMR spectra of dimethyl 5-((4-ethynylphenyl)ethynyl) isophthalate (**1**) in $CDCl_3$.

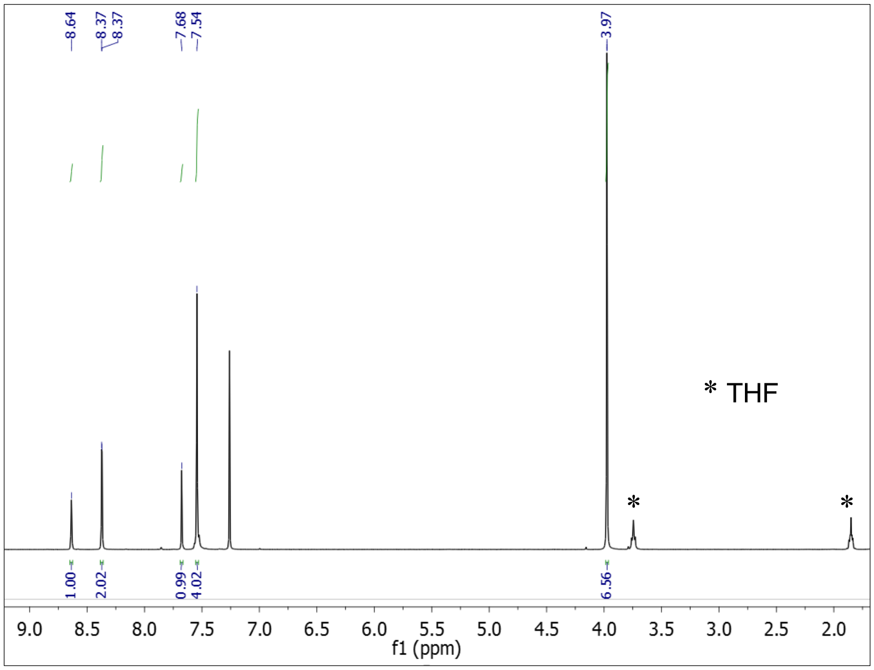

Supplementary Figure 10. ^{1}H (500 MHz) NMR spectrum of 1,3,5-Tris[(1,3-dimethylcarboxylate-5-(4-(ethynyl)phenyl)) ethynyl]benzene (**2**) in $CDCl_3$.

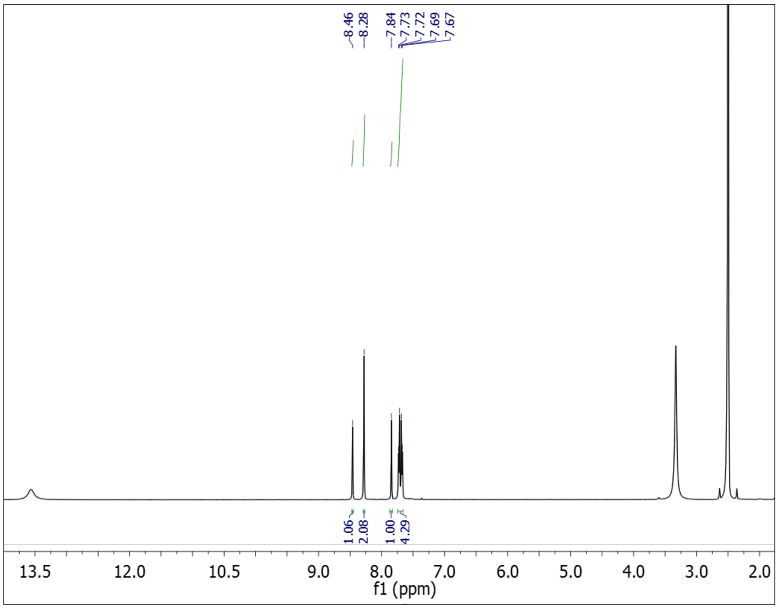

Supplementary Figure 11. ^{1}H (500 MHz) NMR spectrum of 1,3,5-Tris[(1,3-carboxylic acid-5-(4-(ethynyl)phenyl)) ethynyl]benzene (**LH_6**) in DMSO-*d6*.

4.4. Powder X-Ray diffraction (PXRD) patterns

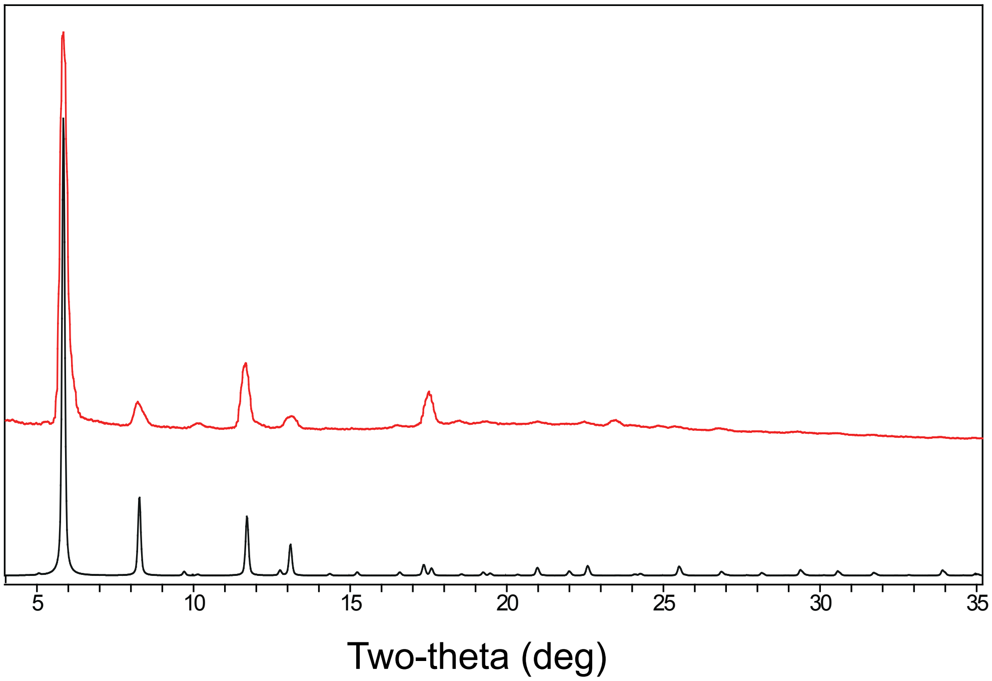

Supplementary Figure 12. The PXRD pattern of SNU-70. Red is the experimental pattern and black is the simulated pattern derived from the single crystal X-ray structure.

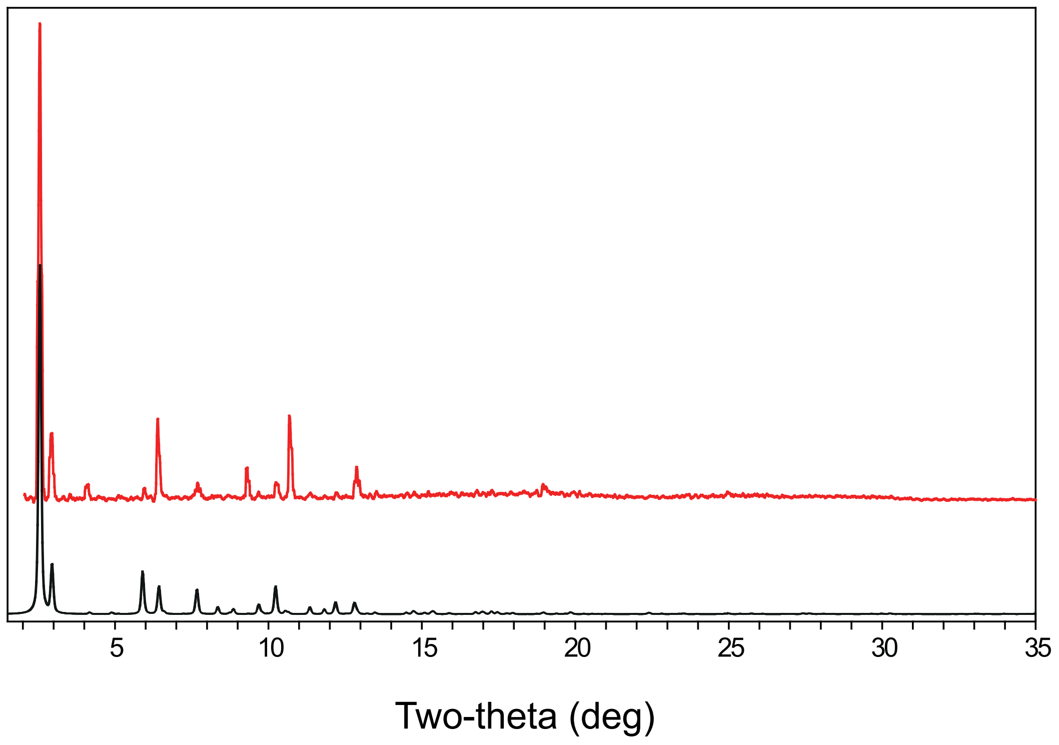

Supplementary Figure 13. The PXRD pattern of NU-100/PCN-610. Red is the experimental pattern and black is the simulated pattern derived from the single crystal X-ray structure.

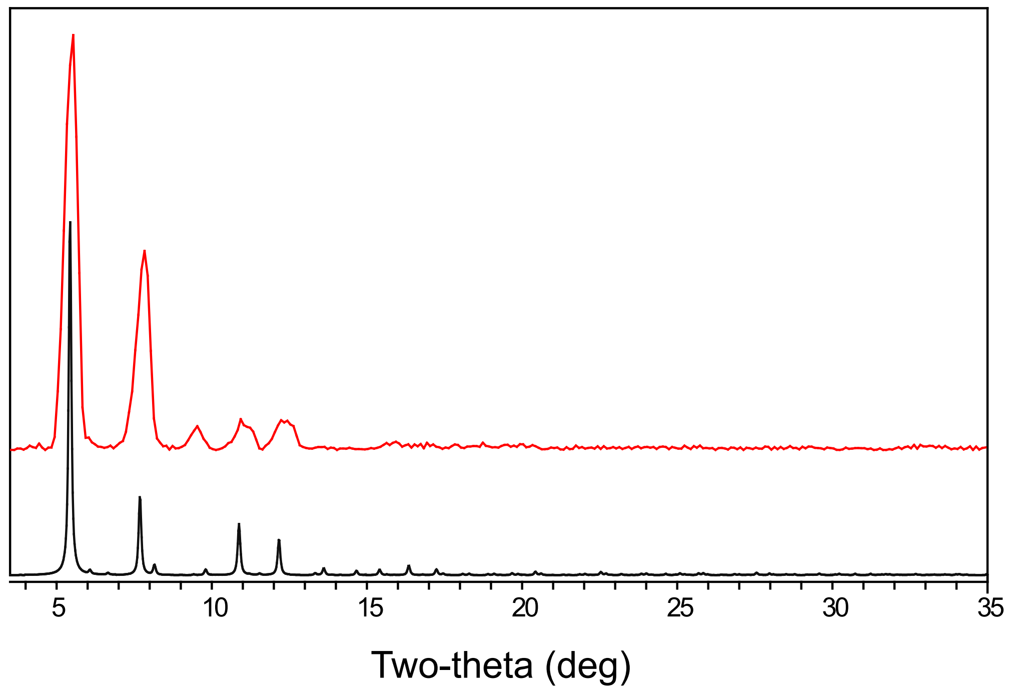

Supplementary Figure 14. The PXRD pattern of UMCM-9. Red is the experimental pattern and black is the simulated pattern derived from the single crystal X-ray structure.

5. Hydrogen uptake measurements

Hydrogen adsorption and desorption measurements were performed using a manometric Sievert's-type instrument (HPVA-200, Micromeritics). The system includes a turbo-molecular pump with an oil-free diaphragm backing pump. The HPVA-2 system was regularly validated at 77 K and room temperature by empty cell measurements and reference material measurements up to 110 bar. The hardware for the commercially available adsorption instrument was unmodified, with the exception of a custom-built stainless steel sample cell. The sample cell connects to a ¼" sample stem by a ½" metal face seal VCR fitting. Two sintered metal filter gaskets are used: a ¼" two micron filter gasket at the top of the sample stem and a ½" five micron filter gasket between the sample stem and sample cell.

MOF samples were loaded in a high-purity argon glovebox, and the sample cell valve was closed off before transferring to the sorption instrument. For activated MOF samples, further degassing was typically not required before measurements unless residual solvent was detected out-gassing from the sample. When required, the degassing procedure for MOFs consisted of heating the sample cell at a low temperature (<100 °C) under continuous vacuum for at least 12 hours.

Void volume measurements were performed using helium at room temperature to estimate both the internal volume of an empty sample cell, and the skeletal density of the samples (to avoid helium adsorption). Because hydrogen adsorption was measured with the sample cell immersed in a liquid N_2 bath, it is necessary to determine the warm and cold void volumes. The warm volume (sub-volume at room temperature) and cold volume (sub-volume at 77 K) of an empty sample cell were measured using helium gas with the liquid N_2 bath filled to a marked level on the sample cell. For subsequent 77 K measurements, the warm and cold void volumes were calculated by subtracting the skeletal volume of the MOF from the empty sample cell volumes. Adsorption and desorption isotherms were measured using the static manometric method with a 5 minute equilibration period for each point. Excess adsorption amounts were calculated from measured pressures and temperatures using a standard mass-balance analysis (which includes the volume displacement of the valve between the sample volume and reservoir volume) along with the current H_2 real gas equation-of-state in REFPROP.[28] Total hydrogen volumetric and gravimetric capacities were calculated following the recommendations in Reference 29 using the MOF crystal density in place of a packing density.

Supplementary References

1 J. Goldsmith, A. G. Wong-Foy, M. J. Cafarella and D. J. Siegel, *Chem. Mater.*, 2013, **25**, 3373–3382.

2 Y. G. Chung, J. Camp, M. Haranczyk, B. J. Sikora, W. Bury, V. Krungleviciute, T. Yildirim, O. K. Farha, D. S. Sholl and R. Q. Snurr, *Chem. Mater.*, 2014, **26**, 6185–6192.

3 P. Z. Moghadam, A. Li, S. B. Wiggin, A. Tao, A. G. P. Maloney, A. Wood, S. C. Ward and D. Fairen-jimenez, *Chem. Mater.*, 2017, **29**, 2618–2625.

4 C. R. Groom, I. J. Bruno, M. P. Lightfoot and S. C. Ward, *Acta Cryst.*, 2016, **72**, 171–179.

5 C. E. Wilmer, M. Leaf, C. Y. Lee, O. K. Farha, B. G. Hauser, J. T. Hupp and R. Q. Snurr, *Nat. Chem.*, 2011, **4**, 83–89.

6 R. L. Martin, C. M. Simon, B. Smit and M. Haranczyk, *J. Am. Chem. Soc.*, 2014, **136**, 5006–5022.

7 Y. Bao, R. L. Martin, C. M. Simon, M. Haranczyk, B. Smit and M. W. Deem, *J. Phys. Chem. C*, 2015, **119**, 186–195.

8 D. A. Gomez-Gualdron, O. V. Gutov, V. Krungleviciute, B. Borah, J. E. Mondloch, J. T. Hupp, T. Yildirim, O. K.

Farha and R. Q. Snurr, *Chem. Mater.*, 2014, **26**, 5632–5639.
9 M. Z. Aghaji, M. Fernandez, P. G. Boyd, T. D. Daff and T. K. Woo, *Eur. J. Inorg. Chem.*, 2016, **2016**, 4505–4511.
10 Y. Bao, R. L. Martin, M. Haranczyk and M. W. Deem, *Phys. Chem. Chem. Phys.*, 2015, **17**, 11962–11973.
11 T. F. Willems, C. H. Rycroft, M. Kazi, J. C. Meza and M. Haranczyk, *Microporous Mesoporous Mater.*, 2012, **149**, 134–141.
12 M. Fischer, F. Hoffmann and M. Fröba, *ChemPhysChem*, 2009, **10**, 2647–2657.
13 R. P. Feynman and A. R. Hibbs, *Quantum mechanics and path integrals*, McGraw-Hill, New York, 1965.
14 A. Ahmed, Y. Liu, J. Purewal, L. D. Tran, M. Veenstra, A. Wong-Foy, A. Matzger and D. Siegel, *Energy Environ. Sci.*, 2017, **10**, 2459–2471.
15 A. K. Rappe, C. J. Casewit, K. S. Colwell, W. A. Goddard and W. M. Skiff, *J. Am. Chem. Soc.*, 1992, **114**, 10024–10035.
16 S. L. Mayo, B. D. Olafson and W. A. Goddard III, *J . Phys. Chem*, 1990, **94**, 8897–8909.
17 D. Dubbeldam, S. Calero, D. E. Ellis and R. Q. Snurr, *Mol. Simul.*, 2016, **42**, 81–101.
18 M. P. Allen and D. J. Tildesley, *Computer simulation of liquids*, Oxford University Press, New York, NY, 1989.
19 R. J. Sadus, *Molecular simulation of fluids: theory, algorithms, and object-orientation.*, Elsevier, Amsterdam, 1999.
20 H. A. Lorentz, *Ann. Phys.*, 1881, **248**, 127–136.
21 S. I. Sandler, *Chemical, biochemical, and engineering thermodynamics*, Wiley, 4th edn., 2006.
22 N. S. Bobbitt, J. Chen and R. Q. Snurr, *J. Phys. Chem. C*, 2016, **120**, 27328–27341.
23 R. L. Martin, L.-C. Lin, K. Jariwala, B. Smit and M. Haranczyk, *J. Phys. Chem. C*, 2013, **117**, 12159–12167.
24 Y. J. Colón, D. A. Gómez-Gualdrón and R. Q. Snurr, *Cryst. Growth Des.*, 2017, **17**, 5801–5810.
25 O. K. Farha, A. Özgür Yazaydın, I. Eryazici, C. D. Malliakas, B. G. Hauser, M. G. Kanatzidis, S. T. Nguyen, R. Q. Snurr and J. T. Hupp, *Nat. Chem.*, 2010, **2**, 944–948.
26 K. Koh, J. D. Van Oosterhout, S. Roy, A. G. Wong-Foy and A. J. Matzger, *Chem. Sci.*, 2012, **3**, 2429.
27 T. K. Prasad and M. P. Suh, *Chem. - A Eur. J.*, 2012, **18**, 8673–8680.
28 E. W. Lemmon, M. L. Huber and M. O. McLinden, NIST Standard Reference Database 23: Reference Fluid Thermodynamic and Transport Properties-REFPROP, Version 10.0, National Institute of Standards and Technology, Standard Reference Data Program, Gaithersburg, 2018.
29 P. A. Parilla, K. Gross, K. Hurst and T. Gennett, *Appl. Phys. A*, 2016, **122**, 201.
