# Supplementary material for: Exceptional hydrogen storage achieved by screening nearly half a million metal-organic frameworks
Source: Nat Commun. 2019 Apr 5;10:1568. doi: 10.1038/s41467-019-09365-w (PMC6450936; doi:10.1038/s41467-019-09365-w)
Supplement: Supplementary file 2 — Peer Review File [file 41467_2019_9365_MOESM2_ESM.pdf]

**Editorial Note:** This manuscript has been previously reviewed at another journal that is not operating a transparent peer review scheme. The manuscript was considered suitable for publication without further review at Nature Communications.
